# Temporal dynamics of gene expression and histone marks at the *Arabidopsis* shoot meristem during flowering

**DOI:** 10.1038/ncomms15120

**Published:** 2017-05-17

**Authors:** Yuan You, Aneta Sawikowska, Manuela Neumann, David Posé, Giovanna Capovilla, Tobias Langenecker, Richard A. Neher, Paweł Krajewski, Markus Schmid

**Affiliations:** 1 Department of Molecular Biology, Max Planck Institute for Developmental Biology, Spemannstrasse 35, 72076 Tübingen, Germany; 2 Department of Biometry and Bioinformatics, Institute of Plant Genetics, Polish Academy of Sciences, Strzeszyńska 34, 60-479 Poznań, Poland; 3 Evolutionary Dynamics and Biophysics Group, Max Planck Institute for Developmental Biology, Spemannstrasse 35, 72076 Tübingen, Germany; 4 Umeå Plant Science Centre, Department of Plant Physiology, Umeå University, SE-901 87 Umeå, Sweden

## Abstract

Plants can produce organs throughout their entire life from pluripotent stem cells located at their growing tip, the shoot apical meristem (SAM). At the time of flowering, the SAM of *Arabidopsis thaliana* switches fate and starts producing flowers instead of leaves. Correct timing of flowering in part determines reproductive success, and is therefore under environmental and endogenous control. How epigenetic regulation contributes to the floral transition has eluded analysis so far, mostly because of the poor accessibility of the SAM. Here we report the temporal dynamics of the chromatin modifications H3K4me3 and H3K27me3 and their correlation with transcriptional changes at the SAM in response to photoperiod-induced flowering. Emphasizing the importance of tissue-specific epigenomic analyses we detect enrichments of chromatin states in the SAM that were not apparent in whole seedlings. Furthermore, our results suggest that regulation of translation might be involved in adjusting meristem function during the induction of flowering.

## Abstract (web summary)

When plants flower, the shoot apical meristem switches fate to produce floral organs instead of leaves. Here You *et al*. perform tissue-specific epigenome profiling and show that during this transition changes in histone methylation are correlated with transcriptional responses in the meristem.

In contrast to most animals, plants have the capacity to produce new organs due to the life-long maintenance of populations of pluripotent stem cell in specialized reservoirs called shoot apical meristems (SAM), from which the aerial parts of a plant, stems, leaves and flowers, are derived^1^. This endows plants with the ability for continued growth, and also allows them to adjust rapidly and flexibly to changes in their environment, which is of particular importance considering the sessile life style of plants^2^. At the time of flowering, the SAM of *Arabidopsis thaliana* undergoes a remarkable phase transition and becomes an inflorescence meristem (IM) that induces on its flanks flower meristems, from which floral organs will differentiate (Supplementary Fig. 1a). At the same time the central IM needs to remain in an undifferentiated state to ensure continued growth^3^.

The correct timing of flowering is crucial to ensure reproductive success and is therefore of both adaptive and economic value^2^. Genetic analyses have identified a number of molecular pathways that perceive and integrate diverse endogenous and environmental signals to ensure that flowering commences under the most favourable conditions^2^,^4^. Prominent examples are the vernalization pathway that regulates flowering in response to prolonged cold (overwintering) and the photoperiod pathway that in *A. thaliana* promotes flowering in long-day (LD) conditions. Day length is perceived in the leaves and through a complex regulatory mechanisms that involves light signalling and the circadian clock^2^ results in the stable expression of the transcription factor (TF) CONSTANS (CO) protein specifically at the end of the LD. CO in turn activates the expression of *FLOWERING LOCUS T* (*FT*) and *TWIN SISTER OF FT* (*TSF*) in the phloem companion cells of the leaf vasculature^5^,^6^. The FT and TSF proteins then act as a long-range signal (florigen) that transduces the information to induce flowering from the leaf to the SAM where they interact with the bZIP TF FD to activate expression of flowering time and floral homeotic genes such as the MADS-box TFs *SUPPRESSOR OF OVEREXPRESSION OF CO 1* (*SOC1*) and *APETALA 1* (*AP1*)^7^.

Most of the known floral regulator genes encode TFs or transcriptional co-regulators that form a complex genetic circuit to ensure flowering is initiated under the right circumstances. While we understand the function of many of the individual genes in this circuit relatively well, genome-wide analyses have been complicated by the fact that flowering time signals are integrated in specific cell types such as the phloem companion cells in leaves and the SAM. Several studies have investigated the transcriptional changes during the initiation of flowering and early flower development at the SAM by either hand dissection or laser-capture micro-dissection of meristems, followed by transcription profiling using either microarrays or RNA-seq^8^,^9^,^10^. These studies provided insights into the changes in expression at the SAM during the floral transition in a tissue-specific manner and identified previously uncharacterized genes with a potential role in flowering time regulation. However, samples generated by these methods are not well suited for epigenomic analyses as they generally contain only small amounts of chromatin. This is unfortunate, as epigenetic factors clearly play an important role in flowering time control^11^. For example, expression of the important flowering time integrator genes *FT*^12^,^13^ in the phloem companion cells of leaves and *FLOWERING LOCUS C* (*FLC*)^14^ in response to vernalization have both been shown to be controlled in part at the chromatin level. However, these studies have been performed on complex tissues or whole seedlings and little is known about the epigenetic processes that regulate flowering time locally, mostly because of the poor accessibility of tissues such as the SAM. It is challenging to isolate enough high quality SAM samples for multiple study approaches in spatio-temporal developmental programs.

To overcome this limitation we have adopted INTACT, which employs *in vivo* biotinylation of nuclear envelopes to facilitate purification of nuclei from specific tissues^15^, to describe the temporal dynamics of the chromatin modifications H3K4me3 and H3K27me3, which have been implicated in promoting and repressing gene expression, respectively^16^,^17^, and their correlation with transcriptional changes at the SAM during photoperiod-induced flowering. Emphasizing the importance of tissue-specific analyses we observe narrow peaks of H3K27me3 close to or even covering the transcription start sites (TSS) of thousands of loci, a chromatin feature rarely observed in epigenomic studies conducted on complex tissues. Our results also suggest that control of translation could be an important factor in regulating meristem function during the induction of flowering. 

## Results

### Establishment of INTACT for the SAM

**Figure 1:**
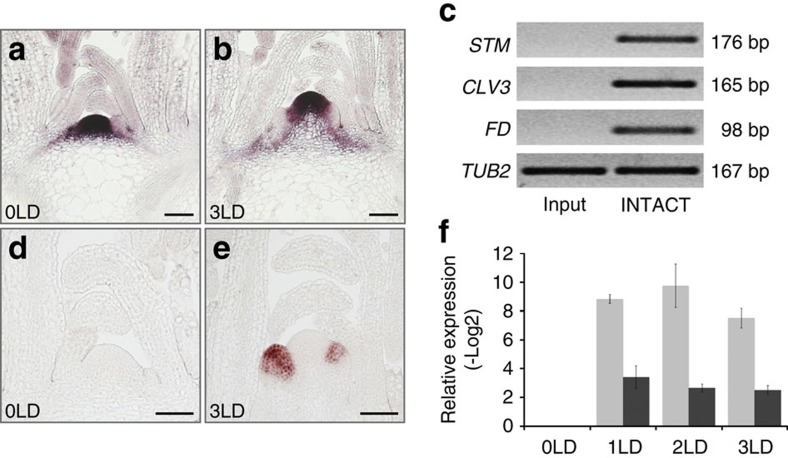
Establishment of the INTACT system for the meristem. (**a**,**b**) Detection of biotinylation and (**d**,**e**) expression of the floral marker gene *AP1* at the meristem of the INTACT line before (**a**,**d**) and 3 days after (**b**,**e**) the shift to LD. Scale bar, 50 μm. (**c**) Semi-quantitative RT–PCR detecting expression of *STM*, *CLV3* and *FD* in the total nuclei from the samples enriched for shoot apical region by manual dissection (input) and in the nuclei isolated by INTACT from the meristem (INTACT). (**f**) Quantitative RT–PCR of *SOC1* (light grey) and *AGL24* (dark grey) in the nuclei from the meristem before (0), and 1, 2 and 3 days after the shift to LD. Error bars, values of two biological replications.

In order to study transcriptional responses and the dynamic of epigenetic modifications at the shoot meristem during the floral transition we first established a modified INTACT reporter line that employs two promoters to biotinylate nuclear envelopes in the entire SAM and IM (Fig. 1a,b and Supplementary Fig. 1c; Methods section^15^,^17^). Enrichment of SAM marker genes such as *SHOOT MERISTEMLESS* (*STM*), *CLAVATA3* (*CLV3*) and *FD* in nuclei isolated from the INTACT line was verified by semi-quantitative RT–PCR (Fig. 1c, Supplementary Figs 1e and 2). Synchronous flowering of the INTACT reporter line was accomplished by shifting 21-day-old plants from non-inductive short days (SD) to inductive LD conditions (Supplementary Fig. 1b). Two to three days after the shift, we consistently detected by RNA *in situ* hybridization robust expression of the flower marker gene *AP1*, indicating floral commitment (Fig. 1d,e and Supplementary Fig. 1d). Furthermore, quantitative reverse transcription PCR (qRT-PCR) using nuclear RNA isolated from meristems of plants transferred from SD to LD demonstrated a significant increase in the expression of the meristematic flowering time integrators *SOC1* and *AGAMOUS-LIKE 24* (*AGL24*) within 24 h after the shift (Fig. 1f). Taken together these initial results indicate that our INTACT line constitutes a system suitable for the study of photoperiod-controlled floral transition at the SAM.

### Differential gene expression at the SAM during flowering

**Figure 2:**
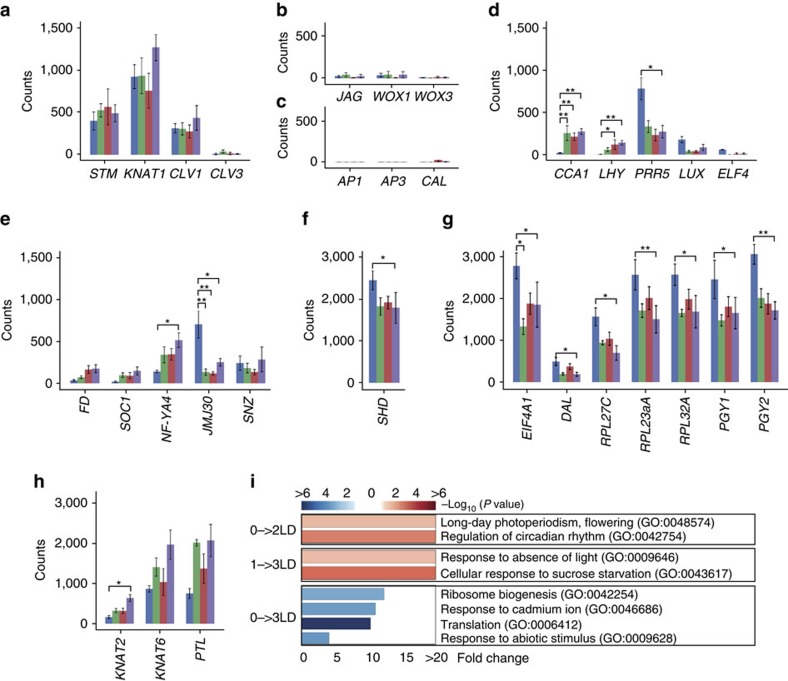
Differential gene expression in the meristem during flowering. (**a**–**h**) Expression (counts of reads) of marker genes for the meristem (**a**), leaf primordia (**b**), flower primordia (**c**), circadian clock (**d**), flowering time (**e**), stem cell (**f**), translation (**g**) and lateral organ boundary (**h**) at the SAM before (blue), and 1 (green), 2 (red) and 3 (purple) days after the shift to LD. Error bars,±s.e. based on four biological replicates; *P<0.05, **P<0.01 (Deseq2 P values corrected for multiple testing using the Benjamini-Hochberg method). (**i**) Biological GO functions enriched for differentially regulated genes with increased (red) and decreased (blue) expression. Only significantly enriched GO terms (*P*<0.05) are reported.

Having established INTACT for the SAM we next performed RNA-seq of the nuclear RNA pool isolated from the meristem for four biological replicates 0, 1, 2 and 3 days after the shift to LD. In total we detected expression (FPKM>1) of ∼60% (19,940) of all genes annotated in *Arabidopsis thaliana* at the SAM at least at one time point (Supplementary Data 1), which is comparable to previous reports^8^,^9^. The meristem identity genes^18^,^19^, *STM, KNOTTED-LIKE FROM ARABIDOPSIS THALIANA 1* (*KNAT1*), *CLAVATA 1* (*CLV1*) and *CLV3* were stably detected in all samples (Fig. 2a), indicating the presence of mRNA originating from meristems and confirming results from the semi-quantitative RT–PCR analysis (Fig. 1c). In contrast, the early floral homeotic genes^20^ *AP1*, *APETALA 3* (*AP3*) and *CAULIFLOWER* (*CAL*), and genes expressed in the centre or abaxial side of developing leaves^21^ such as *JAGGED* (*JAG*), *WUSCHEL RELATED HOMEOBOX 1* (*WOX1*) and *WOX3*, were basically undetectable (Fig. 2b,c), indicating strong enrichment for mRNA from the SAM.

Pairwise comparison at the four time points identified 298 significantly differentially expressed genes, including 148 and 146 genes that were consistently up- or down-regulated, respectively, and 4 genes that exhibited more complex expression patterns (Supplementary Data 2). Interestingly, expression of the morning-expressed genes of the central oscillator of the circadian clock^22^ *LATE ELONGATED HYPOCOTYL* (*LHY*) and *CIRCADIAN CLOCK ASSOCIATED 1* (*CCA1*) was increased significantly 1 day after the shift to LD (Fig. 2d). In contrast, the evening-expressed clock gene^22^ *PSEUDO-RESPONSE REGULATOR 5* (*PRR5*) was significantly down-regulated. *LUX ARRHYTHMO* (*LUX*) and *EARLY FLOWERING 4* (*ELF4*), which encode components of the evening complex, displayed a similar trend as *PRR5*, but the expression changes were not statistically significant after correction for multiple testing (Fig. 2d). These rapid changes in transcript levels most likely reflect a change in the phase of clock gene expression in response to the shift from SD to LD rather than a change in amplitude^23^. Increased expression following the shift to LD was also detected for *SOC1*, *FD* and the CCAAT-binding TF *NF-YA4*, which are known to promote flowering^24^ (Fig. 2e). The expression changes determined by RNA-seq for *SOC1* confirm previous results from qRT-PCR experiments (Fig. 1f), emphasizing the reproducibility of the INTACT method. In addition to induction of floral promoters we also observed decreased expression for the flowering-time repressor *JMJ30* (ref. 25; Fig. 2e). Interestingly, expression of *SHEPHERD* (*SHD*), which is required for stem cell functions and affects the expression of *CLV3* and *WUSCHEL* (*WUS*) and is responsible for the formation of functional CLAVATA proteins^26^, was also significantly decreased upon shift to LD (Fig. 2f). The *shd* mutant is characterized by an increase in stem cell number and an enlarged meristem^26^ and a decrease in *SHD* expression would thus be in agreement with the growth of the meristem that accompanies the floral transition^3^. Taken together these findings indicate that it is possible to capture changes in gene expression of important flowering time and meristem identity genes in the nuclear RNA pool isolated by INTACT from the SAM.

Gene ontology (GO) analysis confirmed that genes related to long-day photoperiodism (GO:0048574) and regulation of circadian rhythm (GO:0042754) were significantly overrepresented among the genes induced at the SAM (Fig. 2i and Supplementary Data 3). In contrast, down-regulated transcripts were significantly enriched for genes related to ribosome biogenesis (GO:0042254) and the control of translation (GO:0006412; Fig. 2i). Affected genes include the translation initiation factor *EIF4A1*, the ribosomal RNA (rRNA) processing factor *DIFFERENTIATION AND GREENING-LIKE* (*DAL*)^27^, and the ribosomal structural constituents *PIGGYBACK 1* (*PGY1)*, *PGY2* and *RPL23aA* (Fig. 2g, Table 1). Interestingly, mutations in the *PGY* genes and *RPL23aA* have previously been shown to cause (or enhance) developmental phenotypes including defects in leaf polarity and inflorescence architecture, and late flowering^28^,^29^,^30^. Taking together these results suggest a connection between the control of translation and the regulation of developmental phase transition at the SAM.

### Changes in H3K4me3 at the SAM correlate with gene expression

To study the correlation between gene expression and chromatin features during floral transition we next analysed the genome-wide distribution of tri-methylation on lysine 4 or 27 of histone 3 (H3), epigenetic marks that are often associated with transcriptional activation (H3K4me3) and repression (H3K27me3), respectively^16^,^17^. ChIP-seq was performed on nuclei isolated from the SAM at the four time points for two biological replicates using H3K4me3- and H3K27me3-specific antibodies (Supplementary Fig. 3).

**Figure 3:**
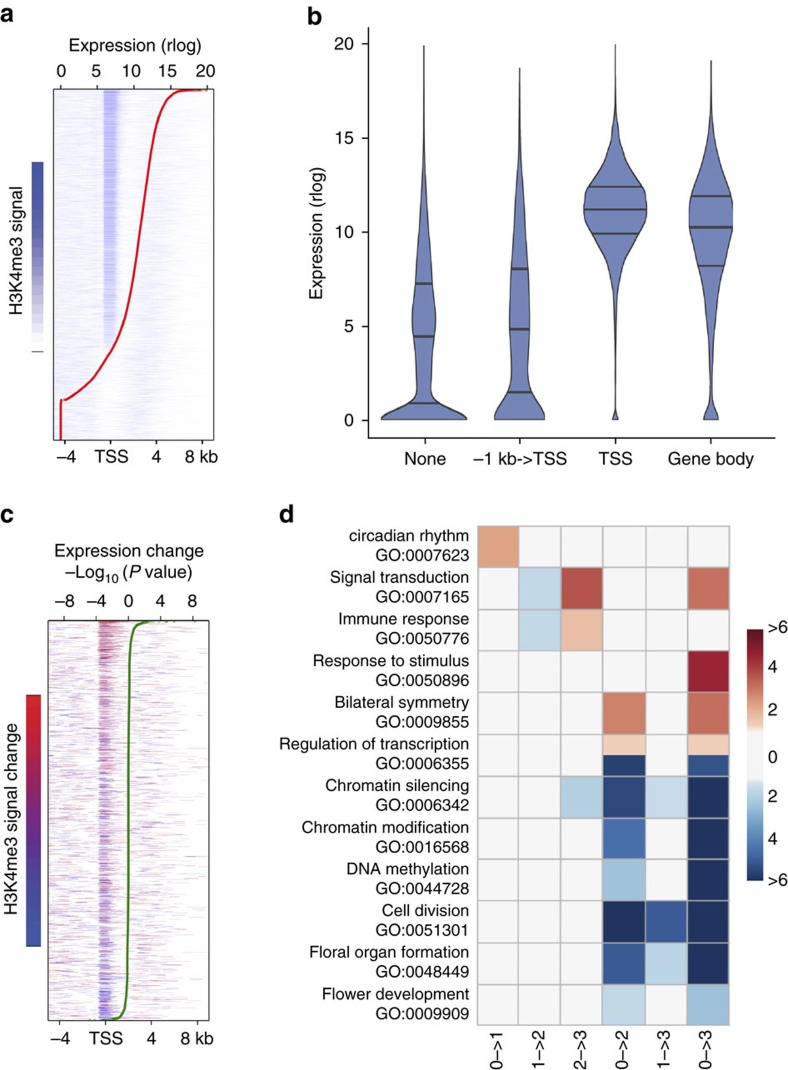
Positive correlation between H3K4me3 and expression. (**a**) Normalized H3K4me3 signal on protein-coding genes in 0 LD samples between −4 kb and +8 kb around the TSS. Genes ordered according to their expression level (regularized logarithm (rlog) of counts, red line). (**b**) Distribution of expression of protein-coding genes in 0 LD samples in relation to the position of the H3K4me3 marks. (**c**) Protein-coding genes with significant differences in H3K4me3 signal between 0 LD and 3 LD samples. Red and blue indicate the direction and significance of gain and loss of H3K4me3, respectively. Genes ordered according to significance (−Log_10_(*P* value)) of changes in expression (green line). (**d**) Key biological processes enriched in genes with significant H3K4me3 changes. Colour key indicates the significance (−Log_10_(*P* value)) of the enriched GO terms that gained (red) and lost (blue) H3K4me3, respectively. Only significantly enriched GO terms (*P*<0.05; fold-change>2) are shown.

**Figure 4:**
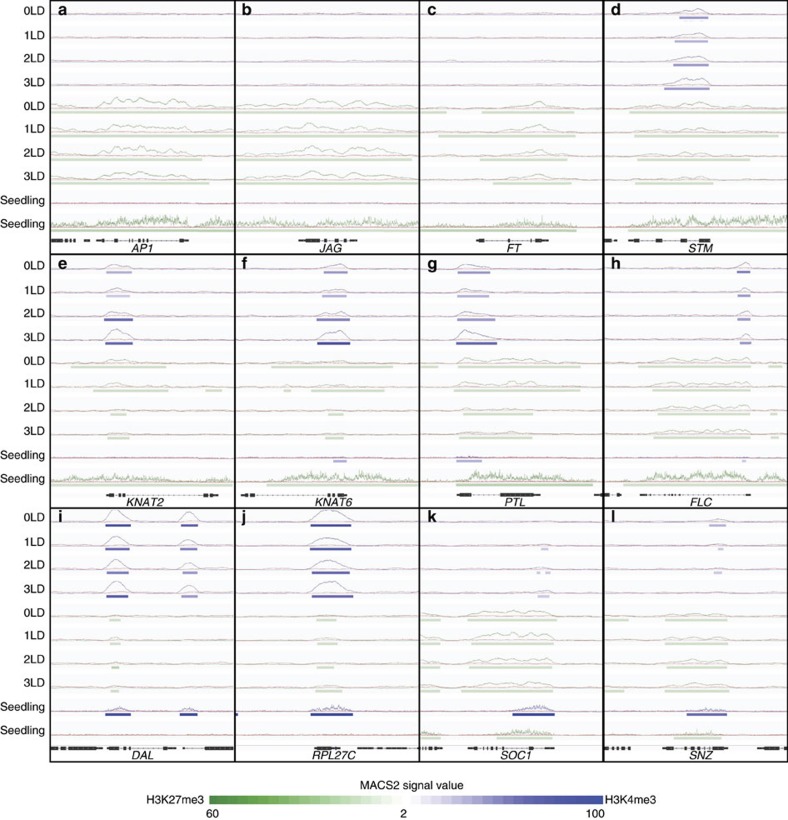
Examples of H3K4me3 and H3K27me3 dynamics at the SAM during the floral transition. (**a**–**i**) Integrative Genomics Viewer traces of *AP1* (**a**), *JAG* (**b**), *FT* (**c**), *STM* (**d**) *KNAT2* (**e**), *KNAT6* (**f**), *PTL* (**g**), *FLC* (**h**), *DAL* (**i**), *RPL27C* (**j**), *SOC1* (**k**), *SNZ* (**l**). H3K4me3, H3K27me3 and H3 signals are depicted in blue, green and red, respectively. Horizontal bars indicate regions and signals of significant enrichment of H3K4me3 and H3K27me3 (according to analysis in MACS2).

H3K4me3 was detected on 61.6% (16,746) of protein-coding genes at least at one time point, and was mostly (81.5%; 13,647) associated with the TSS (Fig. 3a, Supplementary Fig. 4a and Supplementary Data 4). We observed a significant positive association between the presence of H3K4me3 on the TSS and gene expression, as (on average over four time points) the proportion of genes marked with H3K4me3 on the TSS was 5.02% (±0.31%; ±refers to s.e., *n*=4) and 66.13% (± 1.68; ±refers to standard error, *n*=4) for the non-expressed and expressed genes (FPKM>1), respectively (Fig. 3b and Supplementary Fig. 5b). Furthermore, for genes expressed at a given time point, the level of H3K4me3 signal on the TSS was significantly (Spearman rank correlation ranging from 0.29 to 0.33 over the four time points, *P*<0.001, test based on *t* approximation) correlated with the level of gene expression (Fig. 3a and Supplementary Fig. 5a). For example, H3K4me3 was significantly enriched at the TSS of the SAM marker gene *STM* in the SAM samples (Fig. 4d). Over the course of the floral induction, 2,024 genes displayed significant changes in H3K4me3 signal over the gene body at the SAM (Supplementary Data 5). The levels of H3K4me3 changes were positively correlated with the significance of gene expression changes (Fig. 3c and Supplementary Fig. 4b), and 56 genes changed significantly both in H3K4me3 signal and in expression, mostly (89.3%) in the same direction (Supplementary Fig. 6a). The mean expression of genes that increased in H3K4me3 upon shift from SD to LD (pairwise comparisons: 0−>1; 0−>2; and 0−>3) increased significantly (*P*<0.05; test based on rejecting zero slope of linear regression of mean expression against time; Supplementary Fig. 6b), whereas the expression changes observed in the remaining comparisons, while generally following the same trend, were not significant. In summary, our findings suggest a general congruence between H3K4me3 dynamics and gene expression.

GO analysis of the 2,024 genes that exhibited significant changes in H3K4me3 identified 12 overrepresented biological processes (Fig. 3d, Supplementary Data 6). For example, H3K4me3 changed within 24 h after the shift to LDs on genes that form the central oscillator of the circadian clock, as also seen at the transcript level (Fig. 2d), followed by signal receptors, TFs (Supplementary Fig. 7), chromatin remodelers, and categories which play important regulatory roles in cell division, differentiation and developmental phase transitions (Fig. 3d, Supplementary Data 6). Together these data establish changes in H3K4me3 abundance as a fast and important response in the regulation of genes at the SAM during the transition to flowering.

### H3K27me3 is a weak predictor of gene expression at the SAM

**Figure 5:**
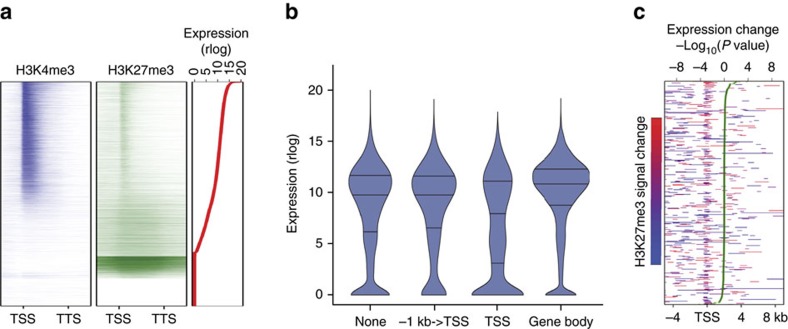
Profiles of H3K4me3 and H3K27me3 marks at the SAM. (**a**) Distribution of normalized H3K4me3 and H3K27me3 signal across the body (±1 kb) of protein-coding genes in 0 LD sample. Regions upstream of the TSS (**−**1 kb) and downstream of TTS (+1 kb) were each divided into 10 bins of length 100 bp. Gene bodies were normalized to account for differences in gene length and divided into 50 bins of equal width. Genes sorted based on levels of expression (red line), and genes with no detectable expression were sorted by H3K27me3 levels. (**b**) Distribution of expression of protein-coding genes in 0 LD samples in relation to the position of the H3K4me27 marks. (**c**) Protein-coding genes with significant differences in H3K27me3 signal between 0 and 3 LDs samples. Red and blue indicate the direction and significance of gain and loss of H3K27me3, respectively. Genes ordered according to significance (−Log_10_(*P* value)) of changes in expression (green line).

Opposing the effect of H3K4me3, which is often enriched at the TSSs of genes and promotes expression (Fig. 3a,b and Supplementary Fig. 5), is H3K27me3, a canonical repressive epigenetic mark^16^,^17^. At the SAM H3K27me3 was detected on 63.69% (17,328) of protein-coding loci at least at one time point (Fig. 5a and Supplementary Data 7). This is in contrast to reports from analyses of complex tissues or whole seedlings, which had reported H3K27me3 on less than 10,000 loci^31^. In agreement with published findings^16^,^17^ broad regions of H3K27me3 were observed on 40.56%±0.95% (average over the four time points; ±refers to s.e., *n*=4) transcriptionally silent genes (FPKM<1, for example Fig. 4a–c). In addition, we also detected an enrichment of H3K27me3 on the TSS of 15.84%±0.93% (average over the four time points; ±refers to s.e., *n*=4) of expressed genes in the SAM (Fig. 5a,b, and Supplementary Fig. 8a,b). The fraction of non-expressed genes (FPKM<1) was increased in the group of genes carrying H3K27m3 mark on the TSS when compared to the other groups (Fig. 5b, Supplementary Fig. 8b). For expressed genes (FPKM>1), the H3K27me3 signal at the TSS was formally negatively correlated with the level of gene expression, though the Spearman rank correlation coefficient was rather low (0.07–0.08 over the four time points, *P*<0.001, test based on *t* approximation). Changes in H3K27me3 at the TSS were not significantly correlated with changes in gene expression between time points (Fig. 5c and Supplementary Fig. 8c). The 472 genes that displayed significant changes in H3K27me3 signal over the gene body during the course of the floral transition (Supplementary Data 8) were not enriched for any biological and molecular functions in GO analysis.

Previous studies had suggested a negative correlation between gene expression level and H3K27me3 abundance^31^,^32^. However, our data indicate that during the transition to flowering changes in gene expression at the SAM are not always reflected in changes in H3K27me3 signals (Fig. 4). For example, the activation of *SOC1* expression in response to LD is accompanied by deposition of H3K4me3 mark whereas H3K27me3 levels remain essentially unchanged (Fig. 4k).

### Opposing H3K4me3-H3K27me3 modification states at the SAM

**Figure 6:**
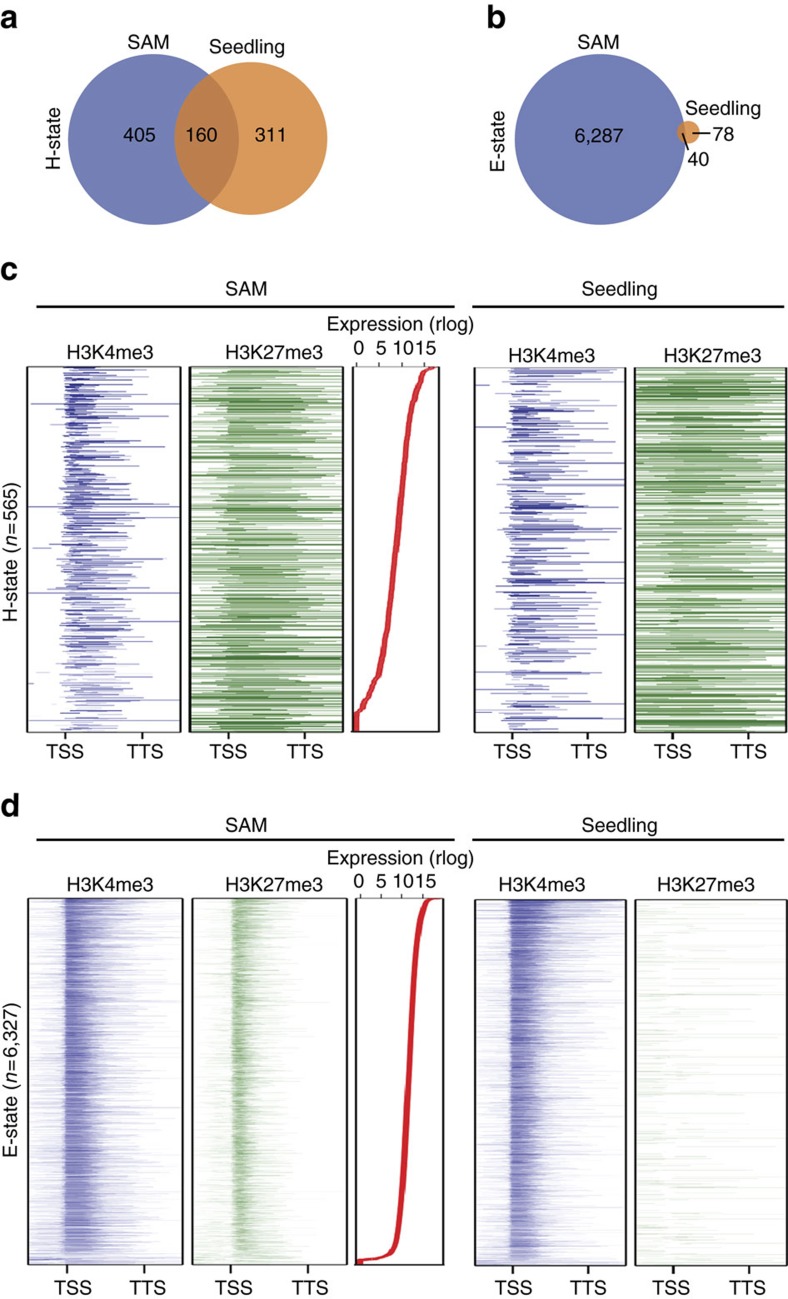
Enrichment of H- and E- states at the SAM. (**a**,**b**) Overlap of genes in H-state (**a**) and E-state (**b**) detected in 0 LD SAM and in whole-seedling samples. (**c**,**d**) Distribution of H3K4me3 and H3K27me3 marks across the gene body (±1 kb) of genes in H-state (**c**) and E-state (**d**) in 0 LD SAM (left) and the whole seedlings (right). Genes were ordered by expression in the meristem samples (red lines).

Overlapping regions of H3K27me3 and H3K4me3 have been described in plants before, both on individual genes as well as in genome-wide analyses^17^,^33^,^34^. In the meristem, co-occurrence of H3K4me3 and H3K27me3 was mainly detected within ±500 bp around the TSS of expressed genes (Fig. 5a; Supplementary Fig. 9a), and two categories of H3K4me3-H3K27me3 states could be distinguished (Fig. 6c,d; Supplementary Fig. 9b, Supplementary Fig. 10, Supplementary Data 9): broad H3K27me3 regions containing a narrow H3K4me3 peak around the TSS (±500 bp), to which we refer as ‘harbouring' state (H-state); and an ‘embedded' state (E-state) with opposite characteristics, that is, narrow H3K27me3 peaks within broader H3K4me3 domains. 28.3% of regions that displayed H-state characteristics in the SAM were also detected in a control data set obtained by performing ChIP-seq in whole seedlings (Fig. 6a,c; Supplementary Data 10). In contrast, the embedded chromatin state was almost exclusively detected in INTACT samples obtained from the SAM and was rarely detected in whole seedlings, demonstrating the power of tissue-specific epigenomic studies (Fig. 6b,d; Supplementary Data 10).

### Functions and dynamics of H- and E- chromatin state genes

**Figure 7:**
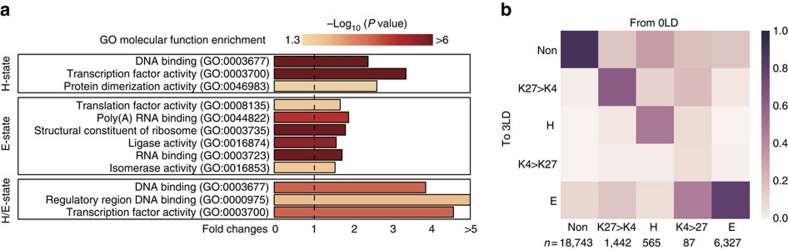
H- and E-state genes at the SAM during floral transition. (**a**) Molecular GO functions enriched for H-state, E-state and H/E-state genes. Only significantly enriched GO terms (*P*<0.05, fold-change>1.5) are reported. (**b**) Matrix of chromatin state transitions from 0–3 LDs for non-overlapping (Non), H-state, and E-state genes, and genes with partial overlapping H3K27me3-H3K4me3 marks (K27>K4; K4>K27). Colours indicate the fraction of genes in a given category on 3 LD, starting from the 0 LD sample (*n*=number of genes in each category in the 0 LD sample).

To gain a better understanding of the potential differences in molecular functions of the H- and E- chromatin states at the TSS we performed GO enrichment analyses (Supplementary Data 11). We found that genes with TF (GO:0003700) and DNA binding activity (GO:0003677) were overrepresented among the genes that were in H-state (Fig. 7a). Over the course of floral induction about half of the H-states present in the vegetative meristem resolved into non-overlapping, partial overlapping or E-states, respectively, demonstrating the dynamic nature of this chromatin state (Fig. 7b). Genes include, for example, the homeobox TFs *KNAT2* and *KNAT6*, and the trihelix TF *PTL* that are involved in meristem and lateral organ boundary development^35^,^36^,^37^. We observed that during floral transition H3K4me3 signal levels for these genes increased, while H3K27me3 domains decreased in length, and that these epigenetic changes were accompanied by increased expression (Fig. 2h). At the end of the floral transition the H-state of *PTL* became partial overlapping (H3K27me3>H3K4me3), and *KNAT2* and *KNAT6* had converted to E-state (Fig. 4e–g).

In contrast, GO term analysis revealed that structural constituents of ribosome (GO:0003735), involved in translation (GO:00081345) and genes that encode for RNA-binding proteins (GO:0003726; GO:0044822) were overrepresented among the E-state genes (Fig. 7a). In this context it is worth remembering that genes involved in ribosome biogenesis, rRNA processing, and in the initiation of translation were significantly enriched among the genes whose expression in the SAM decreased during the transition to flowering (Table 1 and Fig. 2i).

Interestingly, several ribosomal protein genes were reported to express at the highest levels in meristems and young dividing tissues and at the lowest in non-dividing tissues, and mutants of these r-protein genes exhibit multiple developmental lethal/defect phenotypes^38^,^39^,^40^,^41^,^42^. Our result suggested that regulation at the translation level might play important roles for fate decision of the IM, however changes in expression of these translation factors are unrelated with H3K4me3 and H3K27me3 modification levels. Overall, the E-chromatin state was mostly stable (Figs 4i,j and 7b).

## Discussion

Meristems are of interest to plant developmental biology as they harbour stem cells that divide slowly and give rise to daughter cells that eventually differentiate into all other cell types. Despite their importance it is still unclear what exactly distinguishes undifferentiated meristem cells from their differentiated descendants. It seems likely that regulation at the epigenetic and chromatin level contributes to the differentiation of cells derived from the meristem and there is mounting evidence that different cell types exhibit distinct epigenetic features. For example, unique DNA-methylation patterns have recently been reported for different cell types in the root apical meristem^43^. How other epigenetic marks such as acetylation or methylation of histones contribute to the function of meristems and how dynamic these modifications respond to change in the environment is, however, poorly understood.

Our analysis of the dynamic changes of two histone modifications, H3K4me3 and H3K27me3, and their correlation with transcription during the transition to flowering has identified two unusual chromatin states at the SAM. Both of these chromatin states, to which we refer as H- and E-state, display high levels of H3K27me3 at the TSS. In particular the E-state, which is characterized by narrow peaks of H3K27me3 within wider regions of H3K4me3, and which we observe at the four time points analysed in total on more than 9,000 genes stands apart, as H3K27me3 at the TSS has previously been only observed at much lower frequency^33^. Importantly, the E-state might be rather specific to the undifferentiated cells at the SAM as it was not apparent in another INTACT study, which had analysed hair and non-hair cell in the root epidermis^17^, and was also largely absent from the whole-seedling data^33^. Together, these findings provide evidence for SAM-specific chromatin states at the TSS, emphasizing the value of and need for tissue- (or even cell type-) specific epigenomic analyses.

Genes in E-state were significantly enriched for GO categories related to ribosome function and translation. Similarly, ribosomal proteins and translation initiation factors were also overrepresented among the genes whose expression decreased during flowering. Several examples demonstrate the importance of regulation of translation in animal and plant development^42^, and some ribosomal proteins have even been suggested to possess a non-ribosome function^42^,^44^. In *Arabidopsis thaliana*, many ribosomal proteins, including *PGY1*, *PGY2* and *RPL23aA*, are preferentially expressed in meristems and young dividing tissues, and loss of function of these genes causes pleiotropic developmental defects^29^,^30^. Taken together, our results could suggest that control of translational activity might contribute to the regulation of fate transition of the SAM. Finally, RNA-binding proteins that mediate 18S rRNA biogenesis have been shown to be preferentially expressed in mouse embryonic stem cells (ESC) where they play a critical role in sustaining the protein levels of labile pluripotency factors and are required for efficient reprogramming of induced pluripotent stem cells^45^. It is tempting to speculate that, similar to the situation in animal ESCs, control of global translation rates could be involved in maintaining pluripotency of the plant meristem.

Reprogramming of epigenetic states is critical for initial establishment and subsequent maintenance of lineage-specific transcriptional programs during the differentiation of cells. In agreement with the important role of transcriptional reprogramming, we have identified 226 TFs, many of which are known to play important roles in regulation flowering and other developmental processes, among the genes that display significant changes in H3K4me3 at the SAM during the transition to flowering (Supplementary Fig. 7). This includes TFs related to circadian clock function such as *REVEILLE* (*RVE1*)^46^, *CCA1* and *LHY*, whose expression significantly change together with H3K4me3 changes (Supplementary Fig. 7) and that all affect long-day photoperiodism and flowering. However, for most of these TFs we observe only small changes in expression (Supplementary Fig. 11), including the important flowering time regulator *SOC1*, which are not statistically significant after correcting for multiple testing (Supplementary Data 2). Moreover, a few of these TFs do not change in their expression, for example, the flowering time repressor *SCHNARCHZAPFEN* (*SNZ*)^47^, whose H3K4me3 level was significantly decreased at the TSS (Figs 2e and 4l and Supplementary Data 2). Our gene expression data and qRT-PCR experiments support the notion that expression of developmentally regulated genes in the SAM is usually rather low. However, if expression of at least some of these genes were confined to a small sub-population of cells in the SAM this could also result in an underestimation of their actual expression changes. Also we cannot completely rule out the possibility that our low-input RNA-seq method, which involves 2-step cDNA amplification, limits our ability to detect changes of weakly expressed genes. Nevertheless, our results suggest that changes in H3K4me3 are important early events in the regulation of (and might even precede) changes in gene expression at the SAM during the initiation of flowering.

Other known flowering time genes do change significantly in their expression at the SAM in response to LD. One example is *JMJ30*, which encodes a Jumonji-C domain-containing H3K36me2 demethylase^25^. One of the confirmed targets of JMJ30 is *FT*. However, since *FT* is not expressed at the SAM, JMJ30 likely exerts its effect on flowering at the SAM through other targets. *JMJ30* has also been shown to be under direct transcriptional control of CCA1 and LHY, two key components of the central circadian oscillator, which directly bind to *JMJ30* promoter to repress its expression^48^. Loss of *JMJ30* function in turn has been shown to affect the circadian clock^48^. Another example is the CCAAT-motif binding TF, NF-YA4, which has been shown to mediate H3K27me3 demethylation of the *SOC1* promoter to facilitate flowering^49^. In our analysis, expression of *NF-YA4* was significantly decreased at the SAM, however, no changes were observed for H3K27me3 on *SOC1*, which was broadly covered from TSS to TTS throughout the entire experiment (Fig. 4k). Instead, our data suggest that activation of *SOC1* expression is accompanied by increasing H3K4me3 at the TSS (Fig. 4k), similar to what has been described for *FT*, which also is regulated largely independently from H3K27me3 (ref. 50), but strongly affected by H3K4me3 modification level at the TSS^51^.

Interestingly, increased expression of *KNAT2*, *KNAT6* and *PTL*, which are expressed at the boundary between meristem and emerging primordia where pluripotent meristem cells start to differentiate^35^,^36^,^37^, is associated with both increasing H3K4me3 signals and decreasing H3K27me3 coverage. Taking together our results suggest that H3K4me3 has an important role in epigenetic regulated transcription changes at the SAM, whereas the role of H3K27me3 during the early stages of the floral transition is limited.

It remains unclear at this point whether the overlapping H3K4me3 and H3K27me3 signals we observe in our data are a reflection of the sampled SAM tissue, which is composed of different cell types in different stages of differentiation, or if at least some of the peaks are caused by true bivalent marks, that is labelling of a given histone molecule by different modifications. Co-occurrence of H3K27me3 and H3K4me3 has previously been reported, both on individual genes, such as *FT* and *FLC*, as well as in whole-genome analyses^17^,^33^,^34^. These analyses were mostly performed on whole seedlings and the detection of H3K27me3 and H3K4me3 in a specific region of the genome therefore most likely reflects differences in the epigenetic makeup of different cell types rather than bivalent epigenetic marks. However, bivalency does exist in plants as demonstrated by sequential ChIP-PCR analysis in the selected gene loci including *FT* and *FLC*^33^,^34^. At the SAM the *FT* locus was marked broadly with H3K27me3 from TSS to TTS throughout the entire experiment (Fig. 4c) and the *FT* gene was transcriptionally silent (Supplementary Data 1). The *FLC* locus was in H-state (0 LD and 1 LD) or partial overlapping state (H3K27me3>H3K4me3; 2 LD and 3 LD; Fig. 4h, Supplementary Data 9), and no changes were observed for H3K4me3 and H3K27me3 signals nor *FLC* expression.

In ESC, bivalency has been suggested to poise transcription of important differentially regulated genes, but suitable *in vivo* genetic models to probe functions of bivalency in developing organisms are currently missing^52^. Comparisons between ESCs and differentiated cells suggested that bivalent domains tend to resolve during differentiation, which is associated with changes in gene expression^53^. Since the SAM consists of stem cells and only partially differentiated cells, our finding could point towards commonalities in the characteristics of H3K4me3 and H3K27me3 between plants and animals. In our study the H-state is reminiscent of the bivalent situation in ESCs, and TFs were overrepresented among the genes that were in H-state. Unfortunately, sequential ChIP-seq (or -PCR) is at this point not really feasible given the very limited amount of starting material available from INTACT, so that the nature of the overlapping H3K4me3 and H3K27me3 signals at the SAM cannot be answered conclusively at this time.

## Methods

### Oligonucleotides

Sequences of oligonucleotide primers used in this work are listed in Supplementary Table 1.

### Plant growth conditions

*Arabidopsis thaliana* accession Col-0 was used in this work. To synchronize germination and floral transition, all seeds were stratified before sowing in 0.1% agar at 4 °C for 3 day in the dark. Seeds were sown on soil and all plants were grown in growth chambers in a controlled environment, at 23 °C with 65% relative humidity. The light condition was constructed by a mixture of Cool White and Gro-Lux Wide Spectrum fluorescent lights, with a fluence rate of 125–175 μmol m^−2^ s^−1^. LDs were 16 h of light and 8 h of dark, and SDs were 8 h of light and 16 h of dark. In shift experiments for floral induction plants were grown in SDs for 21 days before shifting to LDs.

### Plasmid constructs and the SAM-specific INTACT reporter line

The *E. coli* biotin ligase (BirA) gene was cloned in a modified Gateway entry plasmid pCR8/GW-TOPO (pDP084), and subsequently recombined into a pGREEN-IIS based destination vector (pFK273) that harbours the *Arabidopsis thaliana pUBQ10* (At4g05320) promoter, using a modified Gateway recombination system^54^. The *pUBQ10::BirA* construct (pDP085) was transformed into Col-0 plants making use of *Agrobacterium tumefaciens* strain ASE and the floral dip method^55^. Homozygous *pUBQ10::BirA* lines were identified by selective germination on soil watered with 0.1% glufosinate (BASTA). For INTACT, a modified red nuclear envelope-targeting protein (RedNTF), consisting of the WPP domain (amino acids 1-111) of the *Arabidopsis thaliana* RAN GTPASE ACTIVATING PROTEIN 1 (RanGAP1; At3g63130) at the N terminus, followed by red fluorescent protein (mCherry) for visualization, and the biotin ligase recognition peptide at the C terminus was prepared by PCR using *GL2p:NTF*^17^ as template. The RedNTF open reading frame was cloned in a modified Gateway entry plasmid (pJLSmart) to create pYY1204. For expression of RedNTF at the SAM, two pGREEN-IIS based destination vectors were used. pYY364 consisted of a 1,393 bp promoter fragment of At3g59270, which was cloned in front of the attR1-attR2 GateWay recombination cassette, followed by the At3g59270 terminator sequence (1,404 bp) and drives expression in the entire meristem except for the stem cells. pFK321 consisted of a 1,444 bp promoter sequence of *CLAVATA 3* (*CLV3*) in the front of the attR1-attR2 cassette, followed by the *CLV3* terminator sequence (1,253 bp)^56^ and drives expression of the reporter in the stem cell population at the shoot meristem. *pAt3g59270::RedNTF::tAt3g59270* construct (pYY1208) and *pCLV3p::RedNTF::tCLV3* construct (pYY1301) were generated by recombination of pYY1204 into pYY364 and pFK321, respectively. pYY1208 and pYY1301 were subsequently transformed into homozygous *pUBQ10::BirA* plants to generate *CLV3* and *At3g59270* RedNTF reporter lines. Single insertion lines for the individual RedNTF reporters were identified by selective germination of T2 seeds on ½ MS agar plates containing 50 μg ml^−1^ of kanamycin. INTACT lines that carried both reporters were created by genetic crossings and double-homozygous lines that expressed RedNTF in the meristem were selected by PCR-based genotyping and used in subsequent experiments. PCR reactions were performed using Phusion polymerase or Taq polymerase (New England Biolabs), and all constructs were verified by Sanger sequencing after cloning.

### *In situ* RNA hybridization and Immunohistochemistry

The digoxigenin-labeled anti-sense RNA probe to *APETALA 1* (*AP1*) mRNA was prepared using a published plasmid^57^. Shoot apices from SD-grown plants (21 days) and plants shifted to LD for 1, 2 or 3 days were dissected and fixed in formaldehyde/acetic acid/ethanol (3.7%/5%/50%). Paraplast-embedded material was sectioned to 8 μm thickness and RNA *in situ* hybridization was performed as described^57^. To detect *in vivo* biotinylation by immunohistochemistry sections were dewaxed and rehydrated before applying 1:2,000 diluted streptavidin alkaline phosphatase (Promega) in 1x TBS (0.1% Triton X-100). Permeabilization and immunohistochemical staining were carried out at the room temperature for 1 h 30 min. The signal was developed by a colour reaction using NBT-BCIP solution (Roche).

### Microscopy

A ZEISS Axioplan 2 imaging system was used for imaging the histological sections and for examining the quality and quantity of purified nuclei after each INTACT experiments using DAPI and mCherry filters. In addition, nuclei isolated from the INTACT reporter line were also imaged using a Zeiss LSM510 confocal laser-scanning microscope.

### INTACT purification of SAM nuclei

For each biological replicate, ∼6,000 INTACT reporter plants were grown in SDs for 21 days before shifting to LDs. The 0 LD, 1 LD, 2 LD and 3 LD samples were collected on the 21st day in SDs and 1, 2 and 3 days after the shift to LD. For each sample, the shoot apical region was manually dissected from ∼1,300 INTACT reporter plants and frozen immediately in Eppendorf tubes suspended in liquid nitrogen. Each sample collection took about 3 h from ZT5 to 8 and samples were stored at −80 °C before processing. The INTACT experiments were carried out as described^15^ with the following modifications. Briefly, the 1,300 frozen apices were homogenized to a fine powder with mortar and pestle pre-chilled in liquid nitrogen. Approximately 1/3 of the powder was suspended in 10 ml of ice-cold nuclei purification buffer (NPB: 20 mM MOPS (pH 7), 40 mM NaCl, 90 mM KCl, 2 mM EDTA, 0.5 mM EGTA, 0.5 mM spermidine, 0.2 mM spermine, 1 × Complete protease Inhibitors (Roche)), for isolating non-fixed nuclei by INTACT for transcriptome analysis by RNA-seq. The remaining approximately 2/3 of the ground tissue was suspended in 10 ml of ice-cold NPB buffer containing 1% (vol/vol) formaldehyde (NPBf) and placed under vacuum at RT for 10 min to cross-link proteins to DNAs. The cross-linking was stopped by adding 2 M glycine to a final concentration of 125 mM and incubated for an additional 5 min under vacuum. The fixed nuclei from the SAM were isolated by INTACT and subsequently used to analyze histone modifications by ChIP-seq. Nuclear suspensions were filtered through 40 μm cell strainers (BD Falcon) and pelleted at 1,000*g* for 5 min at 4 °C. Nuclei were washed with 1 ml of NPB, pelleted again by centrifugation and finally resuspended in 1 ml of NPB. For each sample 25 μl of M-280 streptavidin Dynabeads (Invitrogen) were washed with 1 ml of NPB, pelleted at 3,500*g* for 2 min at 4 °C, resuspended in 25 μl NPB, and finally added to the nuclear suspensions. The mixture was rotated at 4 °C for 1 h to allow binding of biotinylated nuclei to the streptavidin-coated beads. The set-up of the INTACT experiments consisted of an OctoMACS TM Seperator (Miltenyi Biotec) placed on ice with BSA-coated 0.6 ml tubes tightly fitted into the grooves. After binding the 1 ml of nuclei-bead suspension was diluted to 10 ml with ice-cold NPB. The 10 ml nuclei-beads suspension of each sample was successively transferred into the 0.6-ml tube fitted in to the OctoMACS TM Seperator, with 3 min intervals in between aliquots to allow nuclei-beads to be captured by the magnet. After each aliquot, the liquid containing un-bound nuclei was removed using a needle and syringe and discarded, until all of the suspension had been processed. The purified nuclei were washed two times with NPB buffer. For each wash step the tubes were carefully removed from the magnets and the magnetic beads were gently resuspended in 500 μl ice-cold NPB and transferred into fresh tubes, which were left at the magnet for 3 min, before the wash buffer was removed using a syringe with a fresh needle and discarded. Finally the purity and the yield of intact nuclei was estimated by microscopy as described^15^ and samples in which INTACT nuclei were enriched to >90% purity were retained and stored at −80 °C before subsequent experiments.

### RNA extraction and double-strand cDNA amplification

Total nuclear RNA was extracted from ∼10,000 non-fixed nuclei of each sample using the RNeasy Micro Kit (Qiagen) following the manufacturers instructions. Contaminating genomic DNA was removed from the RNA samples by treatment with DNase I (0.05 U per μl) for 30 min at 37 °C (Thermo Scientific) before the RNA was purified a second time using the RNeasy Micro Kit (Qiagen). The concentration of the nuclear RNA was determined using the RNA 6,000 Pico Kit (Agilent). The double-stranded cDNA was synthesized from ∼500 pg RNA by linear amplification using the SMARTer Ultra Low Input RNA for Illumina Sequencing-HV kit (Clontech) according to the manufacturer's instructions. The concentration and yield of the amplified cDNA was determined using the High Sensitivity DNA Kit (Agilent).

### Quantitative PCR

The enrichment of the meristematic genes in the isolated nuclei after the INTACT purification was examined by semi-quantitative PCR. ∼100 pg amplified double-stranded cDNA prepared from the input and purified 3 LD samples was used in PCR reactions to examine the expression of *STM*, *CLV3* and *FD* genes (Supplementary Fig. 2) using gene-specific primers (Supplementary Table 1). After 40 cycles the PCR products were separated on a 1.5% agarose gel followed by ethylbromide staining to visualize DNA. Real-time qPCR of *SOC1* and *AGL24* was performed from ∼100 pg amplified double-stranded cDNA, using the Platinum SYBR Green qPCR Supermix-UDG (Invitrogen, Life Technologies) and gene-specific oligonucleotides (Supplementary Table 1) on BioRad C1000 Touch Thermal Cycler. The relative expression values were normalized over the mean expression value of 0 LD samples and calculated by the ΔΔ*C*t method using *TUB2* as control. Because amplification signals from the 0 LD *SOC1* samples did not cross the detection threshold, the *Ct* values was set to 40, the number of cycles used in the PCR programme, to be able to conservatively estimate the differences in expression. Two biological replications with 3 technical replications per sample were used for qPCR analysis.

### RNA sequencing

For transcriptome profiling, 1–2 ng of amplified double-stranded cDNA was sheared to 200–500 bp fragments in water using a focused ultrasonicator (Covaris S2 system), using microtubes at a power of ‘10 dc, 5 i, 200 cpb fs' for 40 s. Subsequently the Low Input Library Prep Kit (Clontech) was used for preparation of sequencing libraries. Libraries were validated using the High Sensitivity DNA Kit (Agilent), and sequencing was performed on an Illumina Hiseq 2,000 system. Approximately 30 million 2 × 101 base-pair paired-end reads that passed the Illumina quality control were collected for each sample in four biological replications (Supplementary Table 2).

### Chromatin immunoprecipitation and sequencing

For ChIP-seq experiments using INTACT-purified nuclei, chromatin was prepared from ∼10,000 purified fixed nuclei per sample as described above. ChIP was performed as described^15^ with the following modifications: chromatin was sheared to 100–500 bp fragments in 200 μl lysis buffer (50 mM Tris, pH 8.0, 10 mM EDTA, 1% SDS, 1 × complete protease inhibitors (Roche)) using a focused ultrasonicator (Covaris S2 system), using microtubes at a power of ‘10 dc, 5 i, 200 cpb fs' for 3 min. The sheared chromatin was divided into three aliquots at the ratio of 1:2:3 for immuno-hybridization with 0.5 μl anti-H3 antibody (Millipore, Cat.17–10254, Lot. 2051404), 1.5 μl anti-H3K4me3 antibody (Millipore, Cat.17–614, Lot.1973237), and 2.5 μl anti-H3K27me3 antibody (Active Motif, Cat. 39155, Lot. 25812014), respectively. The specificity and sensitivity of the antibodies was verified by western blot using nuclear protein extracts as described^58^, and by dot-blot analysis using peptides containing non-, mono-, di- and tri-methylated versions of the respective residues (Abcam, ab2623, ab2903, ab1340, ab7768, ab1342 and ab1780–1782), according to a protocol provided by the manufacturer. Approximately 50 pg of ChIP DNA per sample was isolated using the MinElute Reaction Cleanup Kit (Qiagen). The ChIP-seq libraries were prepared using the ThruPlex-FD Prep Kit (Rubicon Genomics), and linear amplification was monitored by quantitative PCR including SYBR Green I (Invitrogen). Libraries were quantified using the KAPA Library Quantification Kit (KAPABIOSYSTEMS), and further evaluated on a high sensitivity DNA chip (Agilent).

For control ChIP-seq experiments using 3-weeks-old LD-grown seedlings, chromatin was prepared from ∼2 g of fresh material as described^59^. The ChIP experiments were performed as described above and libraries were prepared from ∼5 ng of ChIP DNA using the TruSeq ChIP Library Prep Kit (Illumina). Libraries were quantified on a high sensitivity DNA chip (Agilent).

Libraries were sequenced on Illumina MiSeq, HiSeq 2,000 and HiSeq 3,000 systems for collecting 50 base-pair single-end or 150 base-pair paired-end reads to high sequencing depth^60^ (Supplementary Table 2). Only Read 1 of 150 base-pair paired-end reads were used and further trimmed to 50 base pair. Uniquely mapped 50 base-pair single-end reads were used for ChIP-seq analyses.

### RNA-seq data analysis

Next generation sequencing (NGS) reads were mapped to *Arabidopsis thaliana* reference transcriptome TAIR10 ver. 24, with ribosomal RNA regions (2:3471–9557; 3:14,197,350–14,203,988) masked, using TopHat 2.0.13 (no-mixed alignments; up to 20 secondary alignments; no novel junctions)^61^. Counts of NGS reads covering transcripts were computed using the function summarizeOverlaps^62^ in R. Expressed genes were defined as those having the value of FPKM>1 at least at one time point. Read counts were submitted to differential gene expression analysis in Deseq2 (default parameters, FDR<0.05)^63^. Regularized logarithms of read count computed by Deseq2, denoted by *rlog*, were used for the analysis of relationships between gene expression level and histone modifications signal.

### Histone modification data analysis

NGS reads were mapped to *Arabidopsis thaliana* reference genome TAIR10 ver. 24 using Bowtie2 2.2.4 (minimum alignment score −12.5; uniquely mapped reads only)^64^. The sets of aligned H3K27me3 and H3 reads were down-sampled to minimum number of reads, respectively, for peak-calling from the same sequencing depth in all samples^65^. Significant enrichment regions in H3K4me3 and H3K27me3 samples, relative to H3 control samples, were identified using MACS2 2.1.0 (default settings for H3K4me3—FDR<0.05; broad-peaks mode for H3K27me3—FDR<0.1)^66^,^67^. Regions with signal level measured by FPKM(ChIP)/FPKM(H3 control) differing between biological replications by more than 2 s.d. were filtered out. Consensus regions found as a sum of regions from two replications were taken as histone modification marks. Differential modification analysis for H3K4me3 and H3K27me3 marks was performed using DiffBind^68^ (Bioconductor 3.2; minoverlap=2; FDR<0.01). The modification marks were intersected with annotated genes to obtain lists of genes with specific coverage by H3K4me3 and H3K27me3. Spearman rank correlation coefficients were computed and tested for significance in Genstat 18 (ref. 69).

### GO term analysis

AmiGO 2 versions 2.3.2 and 2.4.24 (ref. 70) were used for GO analysis to assess the over- and underrepresentation in biological processes of significantly differentially-H3K4me3-modified genes and molecular functions of H-state and E-state genes. *P* values were corrected for multiple hypothesis testing (Bonferroni correction), and only *P*<0.05 were considered as significant. To reduce the complexity redundant child terms based on GO hierarchy were removed from the reports.

### Data availability

ChIP-seq and RNA-seq data have been deposited with ArrayExpress database (www.ebi.ac.uk/arrayexpress), accession numbers E-MTAB-4680, E-MTAB-4684 and E-MTAB-5130. The authors declare that all other data supporting the findings of this study are available within the manuscript and its supplementary files or are available from the corresponding author upon request.

## Additional information

**How to cite this article:** You, Y. *et al*. Temporal dynamics of gene expression and histone marks at the *Arabidopsis* shoot meristem during flowering. *Nat. Commun.* **8,** 15120 doi: 10.1038/ncomms15120 (2017).

**Publisher's note**: Springer Nature remains neutral with regard to jurisdictional claims in published maps and institutional affiliations.

## Supplementary Material

Supplementary Information Supplementary Figures and Supplementary Tables

Supplementary Data 1 Gene expression profiles at the meristems.

Supplementary Data 2 Differentially expressed genes during floral transition.

Supplementary Data 3 GO enrichment in biological process of significantly differentially-expressed genes.

Supplementary Data 4 H3K4me3 modifications at the four time points.

Supplementary Data 5 Genes with significant H3K4me3 signal changes in pair-wise comparisons between 0LD, 1LD, 2LD and 3LD samples.

Supplementary Data 6 GO enrichment in biological process of significantly differentially-H3K4me3-modified genes.

Supplementary Data 7 H3K27me3 modifications at the four time points.

Supplementary Data 8 Genes with significant H3K27me3 signal changes in pair-wise comparison between 0LD, 1LD, 2LD and 3LD samples

Supplementary Data 9 Categories of potentially bivalent chromatin states on protein coding genes.

Supplementary Data 10 H3K4me3 and H3K27me3 modifications in whole seedling samples.

Supplementary Data 11 GO enrichments in molecular functions of H-state, E-state, and H/E-state genes.

## Figures and Tables

**Table 1:** Significantly differentially expressed structural constituents of ribosome and genes for RNA-binding proteins.

**ID**	**Gene**	**Expression change**	Chromatin state*	**Annotation**
AT1G01100	*RPP1A*	Dec	E	60S acidic ribosomal protein P1-1
AT1G15930	*RPS12A*	Dec	E	40S ribosomal protein S12-1
AT1G41880	*RPL35AB*	Dec	E	60S ribosomal protein L35a-2
AT2G19730	*RPL28A*	Dec	E	60S ribosomal protein L28-1
AT2G36160	*RPS14A*	Dec	E	40S ribosomal protein S14-1
AT2G39460	*ATRPL23A*	Dec	E	60S ribosomal protein L23a-1
AT3G13920	*EIF4A1*	Dec	E	Eukaryotic initiation factor 4A-1
AT3G22230	*RPL27B*	Dec	E	60S ribosomal protein L27-2
AT3G49910	*RPL26A*	Dec	E	60S ribosomal protein L26-1
AT3G53460	*CP29*	Dec	E	29 kDa ribonucleoprotein, chloroplastic
AT3G53740	*RPL36B*	Dec	E	60S ribosomal protein L36-2
AT4G15000	*RPL27C*	Dec	E	60S ribosomal protein L27-3
AT4G16390	*SVR7*	Dec	E	Pentatricopeptide repeat-containing protein,
AT4G18100	*RPL32A*	Dec	E	60S ribosomal protein L32-1
AT4G30930	*NFD1*	Dec	E	50S ribosomal protein L21, mitochondrial
AT5G02870	*RPL4D*	Dec	E	60S ribosomal protein L4-2
AT5G16130	*RPS7C*	Dec	E	40S ribosomal protein S7-3
AT5G18380	*RPS16C*	Dec	E	40S ribosomal protein S16-3
AT5G23740	*RPS11-BETA*	Dec	E	40S ribosomal protein S11-3
AT5G27850	*RPL18C*	Dec	E	60S ribosomal protein L18-3
AT3G02830	*ZFN1*	Inc	E	Zinc finger CCCH domain-containing protein 33
AT1G33120	*RPL9B*	Dec	None	60S ribosomal protein L9-1
AT1G33140	*PGY2*	Dec	None	60S ribosomal protein L9-1
AT2G27530	*PGY1*	Dec	None	60S ribosomal protein L10a-2
AT3G55750	*RPL35AD*	Dec	None	60S ribosomal protein L35a-4
AT3G60770	*RPS13A*	Dec	None	40S ribosomal protein S13-1
AT3G62250	*UBQ5*	Dec	None	Ubiquitin-40S ribosomal protein S27a-3
AT3G13740	*MMM17.15*	Dec/Inc	None	Ribonuclease III family protein
AT3G12915		Inc	None	Ribosomal protein S5/Elongation factor G/III/V

^*^Genes that were in H-state, E-state (E) or none of the two (None) at least at one time point.
