# Supplementary material for: Temporal dynamics of gene expression and histone marks at the Arabidopsis shoot meristem during flowering
Source: Nat Commun. 2017 May 17;8:15120. doi: 10.1038/ncomms15120 (PMC5442315; doi:10.1038/ncomms15120)
Supplement: Supplementary Information — Supplementary Figures and Supplementary Tables [file ncomms15120-s1.pdf]

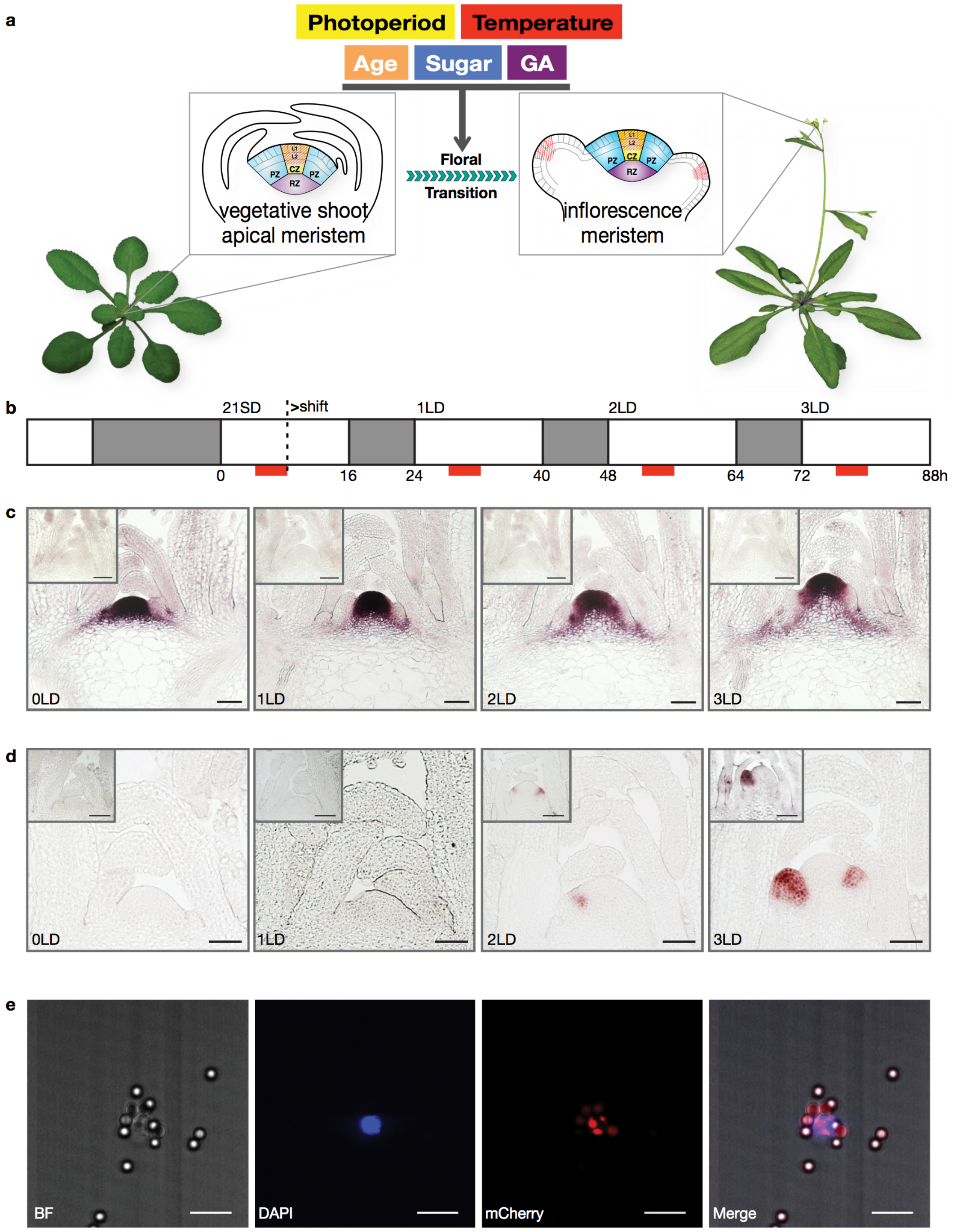

Supplementary Figure 1. Synchronous floral-transition in the INTACT reporter line. a, Reorganization of the shoot apical meristem during floral transition. The central zone (CZ), peripheral zone (PZ) and rib zone (RZ) are indicated in yellow, blue, and purple, respectively. The position of the stem cells is hatched. **b,** Time course of floral transition experiment. Plants were grown for 21 short days (SD) and transferred to 3 consecutive long days (LD) to induce flowering. Light and dark hours are indicated in white and grey, respectively. The time of sample collection (Zeitgeber 5-8) is indicated with a red bar. **c-d,** Detection of biotinylation (**c**) and expression of the floral marker gene *AP1* (**d**) at the meristem of the INTACT reporter line and wild-type Col-0 controls (inserts), before (0LD) and 1, 2, and 3 days after the shift to LD. Scale bar, 50 µm. **e,** Fluorescence microscopy images of a nucleus purified from the INTACT reporter line. Magnetic beads show as white spheres in bright field (BF), DNA stained with DAPI is shown in blue, and nuclear envelope domains tagged with RedNTF are detected by mCherry fluorescence. Scale bar, 10 µm.

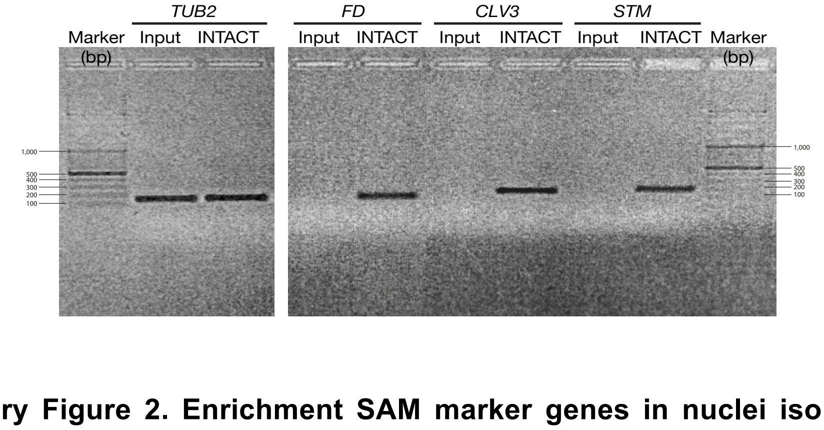

Supplementary Figure 2. Enrichment SAM marker genes in nuclei isolated from the meristem INTACT reporter line. Semi-quantitative RT–PCR detecting expression of *STM*, *CLV3,* and *FD* in nuclei from samples enriched for shoot apices by manual dissection (Input) and in nuclei isolated by INTACT from the meristem (INTACT).

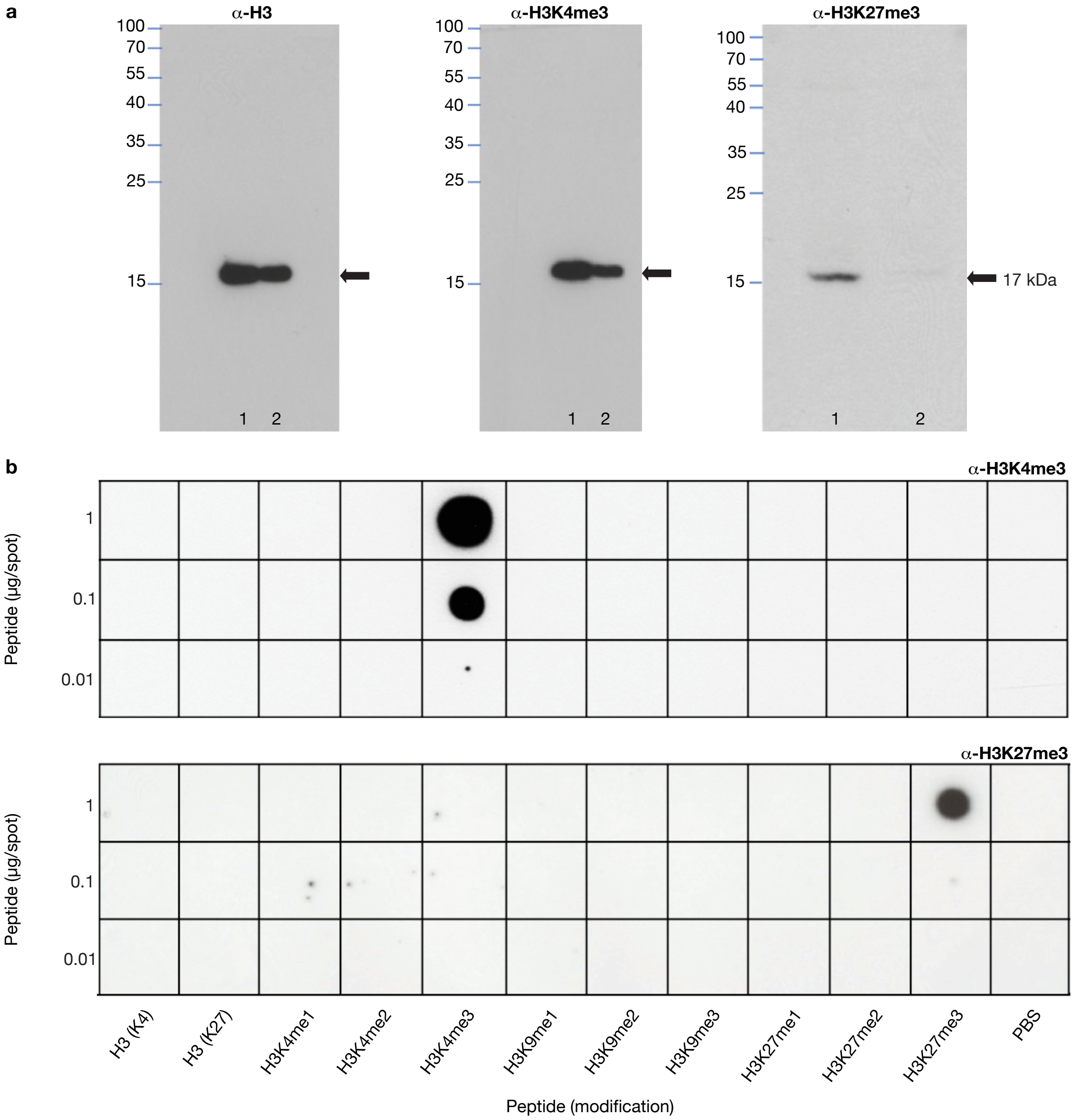

Supplementary Figure 3. Validation of the antibodies used for ChIP. a, Western blot analyses of nuclear protein extracts of inflorescence tissues (Lane 1) and from nuclei used as input for INTACT (Lane 2) using antibodies against H3, H3K4me3, and H3K27me3. **b**, Verification of antibody specificity by dot blot analysis using peptides with non-, mono-, di-, and tri-methylated K4 and K27 residues of H3.

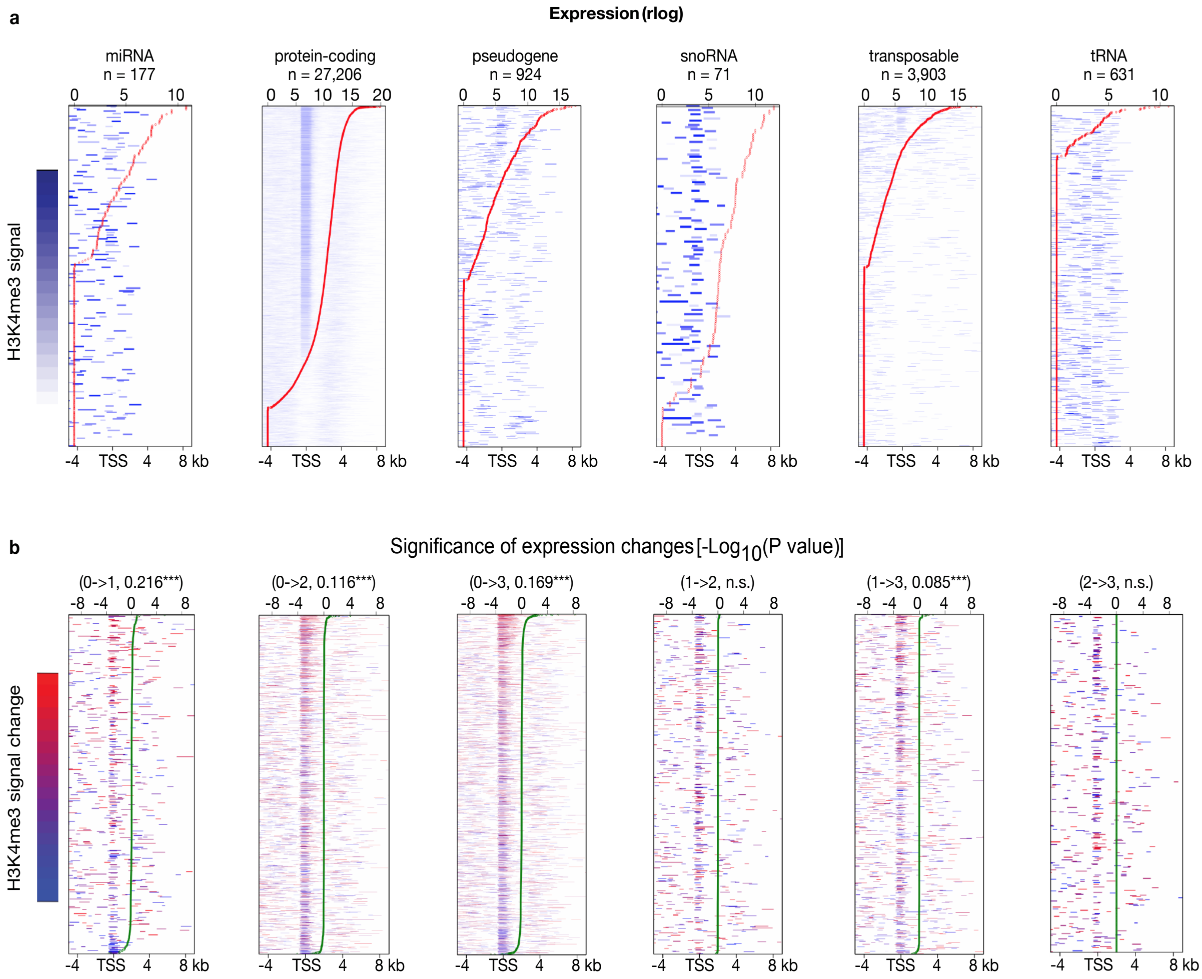

Supplementary Figure 4. Profiles of H3K4me3 modification at the SAM during floral transition. a, Normalized H3K4me3 signal on annotated genes (TAIR10) in 0LD samples between -4 kb to +8 kb around the TSS. Genes of different functional categories were ordered by their expression level [regularized logarithm (rlog) of counts, red line]. ncRNAs and categories with few genes (rRNA, n=4; snRNA, n=24) were excluded. Color intensity designates the normalized signal (RPKM(H3K4me3)/RPKM(H3); mean value of two biological replicates) of H3K4me3. **b**, Protein-coding genes with significant differences in H3K4me3 signal in pair-wise comparisons between 0LD, 1LD, 2LD and 3LD samples. Genes ordered according to significance (-Log$_{10}$(P value)) of changes in expression (green line). Red and blue indicate the direction and significance of gain and loss of H3K4me3, respectively. The rank correlation coefficient between expression change and H3K4me3 modification change is shown above the graph.

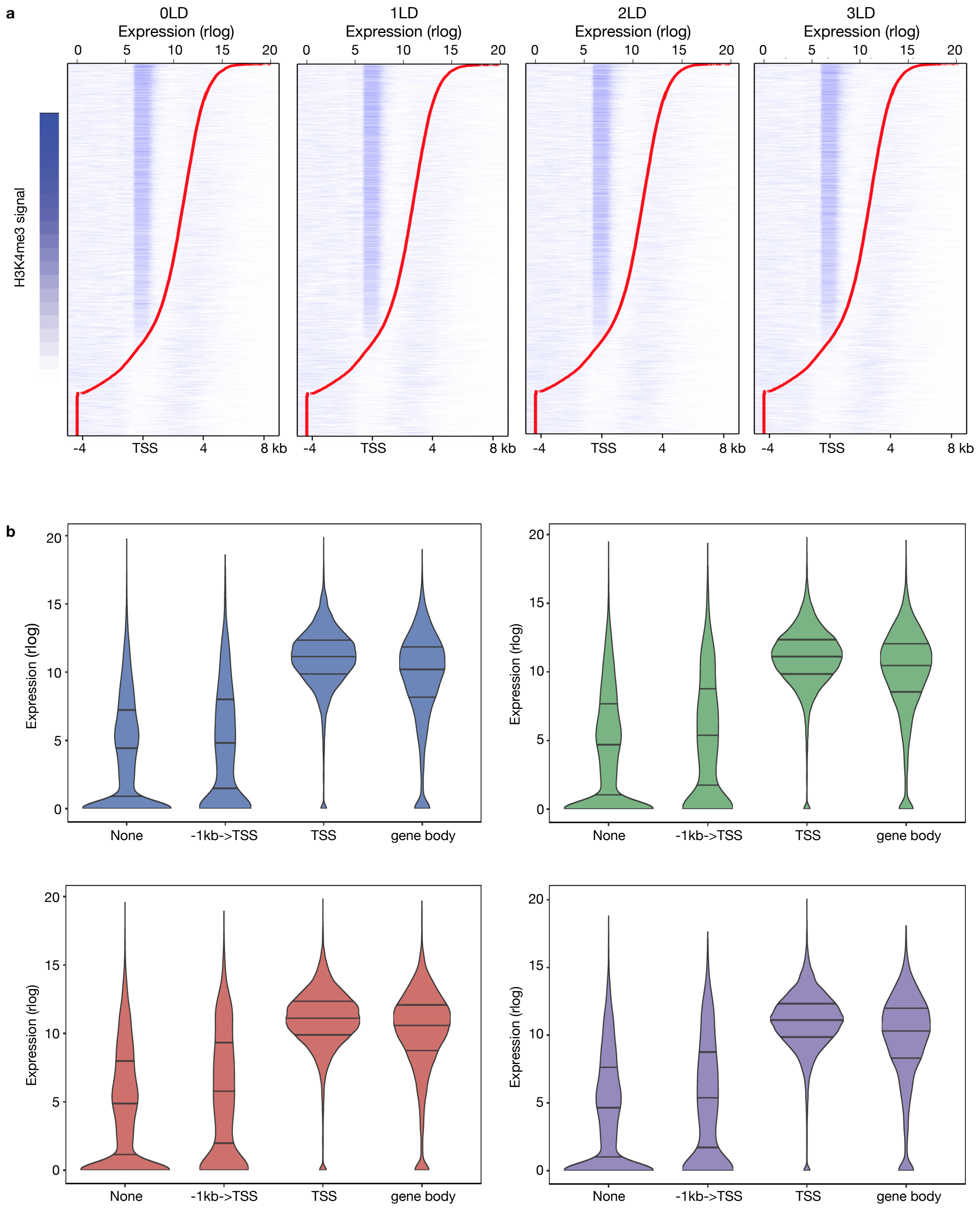

Supplementary Figure 5. H3K4me3 marks at the TSS are positively correlated with expression of protein coding genes. a, Normalized H3K4me3 signal on protein-coding genes between -4 kb to +8 kb around the TSS at the four time points. Genes ordered according to their expression levels [regularized logarithm (rlog) of counts, red line]. Color intensity designates the normalized (RPKM(H3K4me3)/RPKM(H3); mean value of two biological replicates) signal of H3K4me3. **b**, Distribution of expression [regularized logarithm (rlog) of counts] of protein-coding genes from the SAM before (blue), and 1 (green), 2 (red), and 3 (purple) days after the shift to LD in relation to the position of the H3K4me3 peak on the gene.

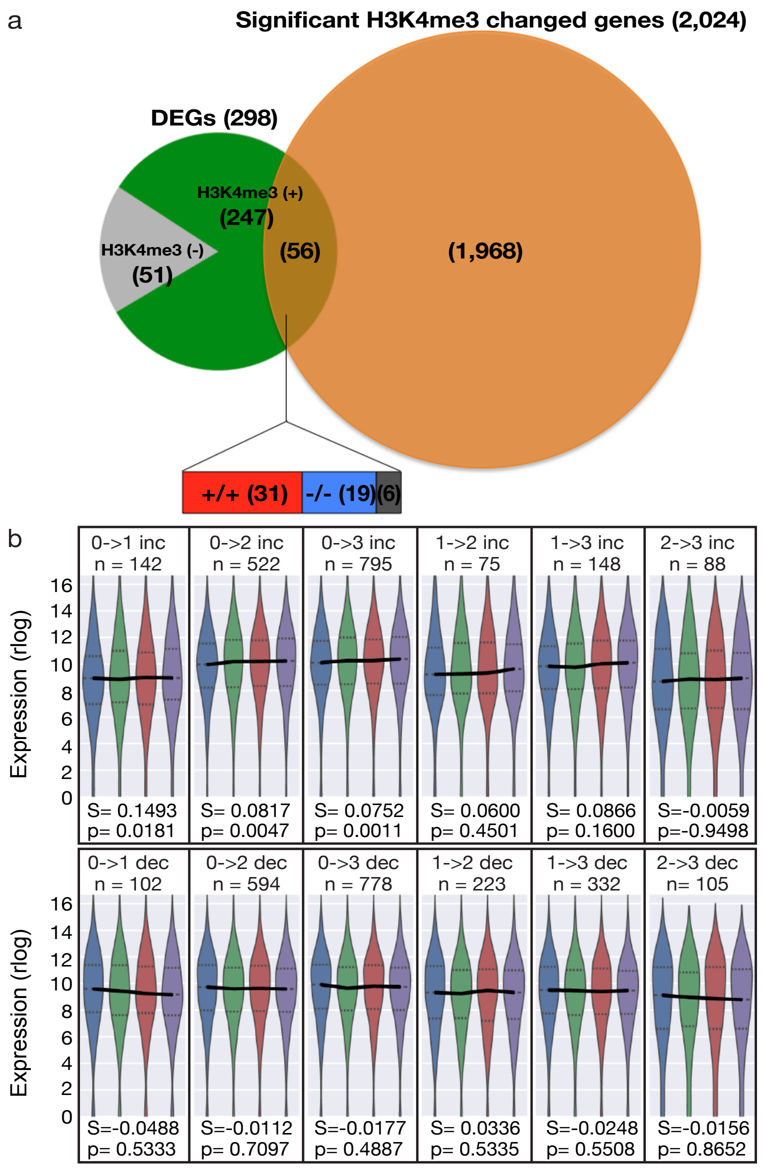

Supplementary Figure 6. Genes with significant H3K4me3 signal changes during floral transition. a, Overlap between statistically differentially expressed genes (DEGs; 298) and genes with significant H3K4me3 changes (2,024). DEGs that were (247) or were not (51) marked with H3K4me3 at least at one time point are shown in green and grey, respectively. 56 genes changed in expression as well as in H3K4me3. Of the 56 genes changed in expression as well as in H3K4me3, 31 (red) increased, 19 (blue) decreased, and 6 (dark grey) were mixed-regulated. **b**, Correlation between H3K4me3 and expression for 1,968 non-significantly differentially expressed genes with significant H3K4me3 changes. Individual panels display the distribution of expression [regularized logarithm (rlog) of counts] of genes with significant changes in H3K4me3 (n) for a given pairwise comparison (0->1; 0->2; etc.) at the four time points analyzed [blue: 0LD (=SD); green: 1LD; red: 2LD; purple: 3LD]. Genes with increased (inc) and decreased (dec) H3K4me3 are shown in the top and bottom row, respectively. The median of gene expression is shown by a bold line. The slope (S) of a linear regression of expression vs. time point is shown below each panel along with the associated p-value.

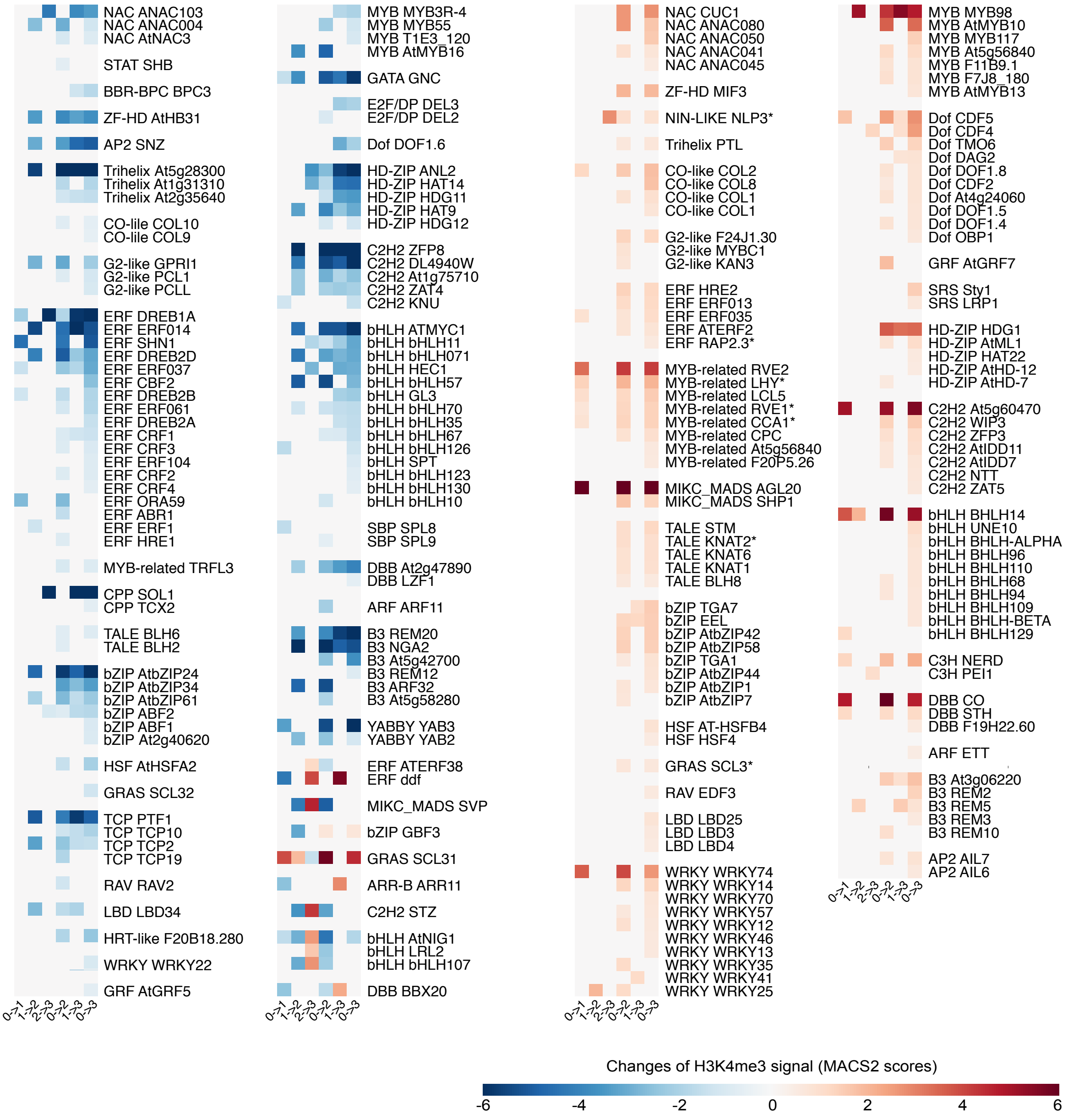

Supplementary Figure 7. Groups of transcription factors that are differentially modified with H3K4me3 marks at the SAM during floral transition. Red and blue indicate the direction and values of gain and loss of H3K4me3 signals, respectively. Genes with significant change in expression are indicated with *.

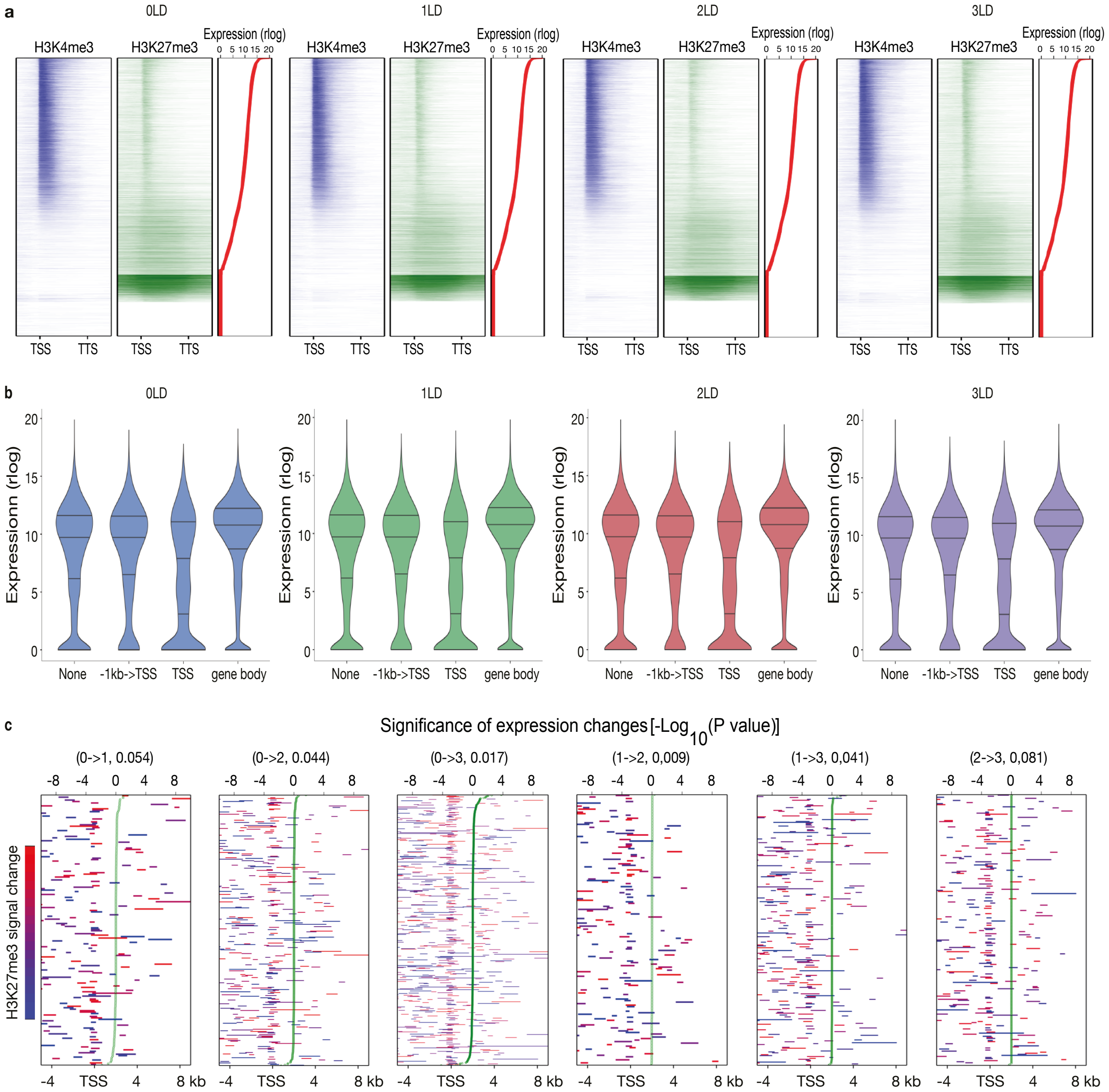

Supplementary Figure 8. Profiles of H3K4me3 and H3K27me3 modification in the SAM at the four time points. **a**, Distribution of H3K4me3 and H3K27me3 marks across the body (±1 kb) of protein-coding genes in the meristems at the four time points. Regions upstream of the TSS (-1 kb) and downstream of TTS (+1 kb) were each divided into 10 bins of length 100 bp. Gene bodies were normalized to account for differences in gene length and divided into 50 bins of equal width. Genes were sorted based on levels of expression [regularized logarithm (rlog) of counts, red line], and genes with no detectable expression were sorted by H3K27me3 levels. **b**, Distribution of expression [regularized logarithm (rlog) of counts] of protein-coding genes from the SAM before (blue), 1 (green), 2 (red), and 3 (purple) days after the shift to LD in relation to the position of the H3K27me3 peak on the gene. **c**, Protein-coding genes with significant differences in H3K27me3 signal in pair-wise comparisons between 0LD, 1LD, 2LD, and 3LD samples. Red and blue indicate the direction and significance of gain and loss of H3K27me3, respectively. Genes ordered according to significance ($-Log_{10}$(P value)) of changes in expression (green line). The rank correlation coefficient between expression change and H3K27me3 signal change is shown above the graph.

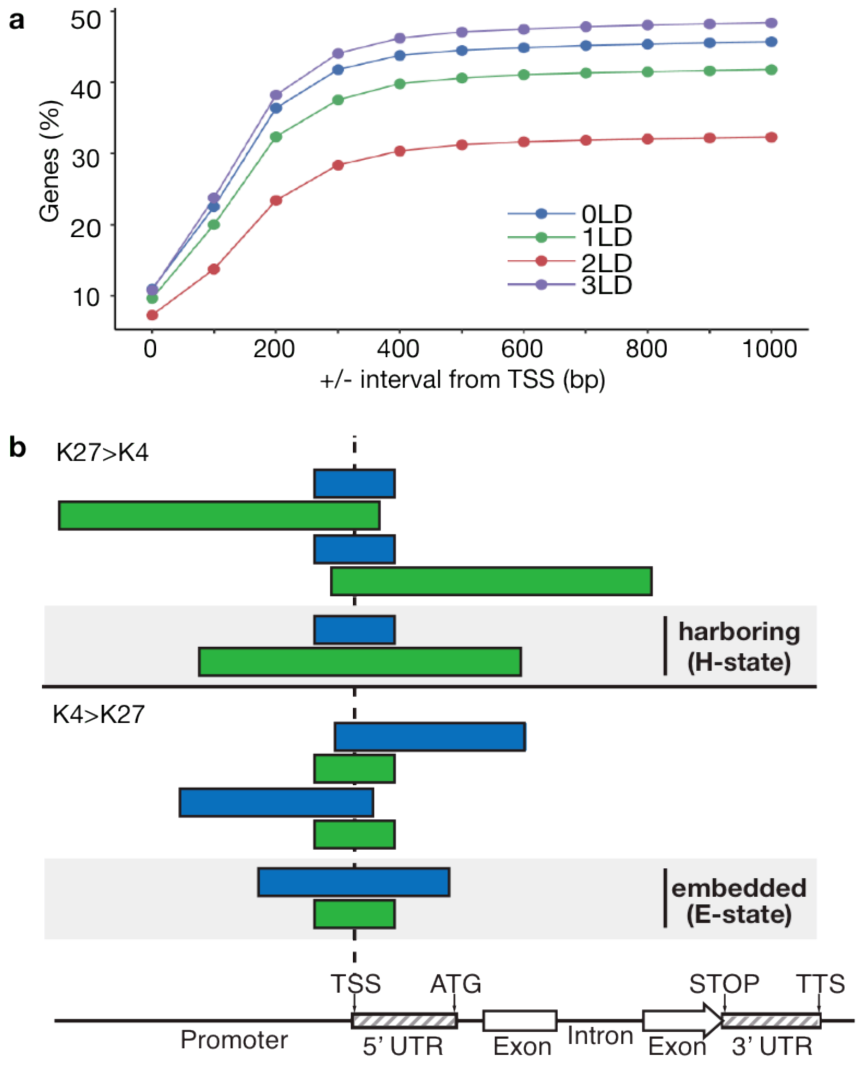

Supplementary Figure 9. Correlation between distance from TSS and co-occurrence of H3K4me3 and H3K27me. a. Percentage of expressed (FPKM>1) protein-coding genes with overlapping H3K4me3 and H3K27me3 depending on the size of the interval around the TSS. **b**, Graphic representation of overlapping states of H3K4me3 (blue) and H3K27me3 (green) marks in the meristem.

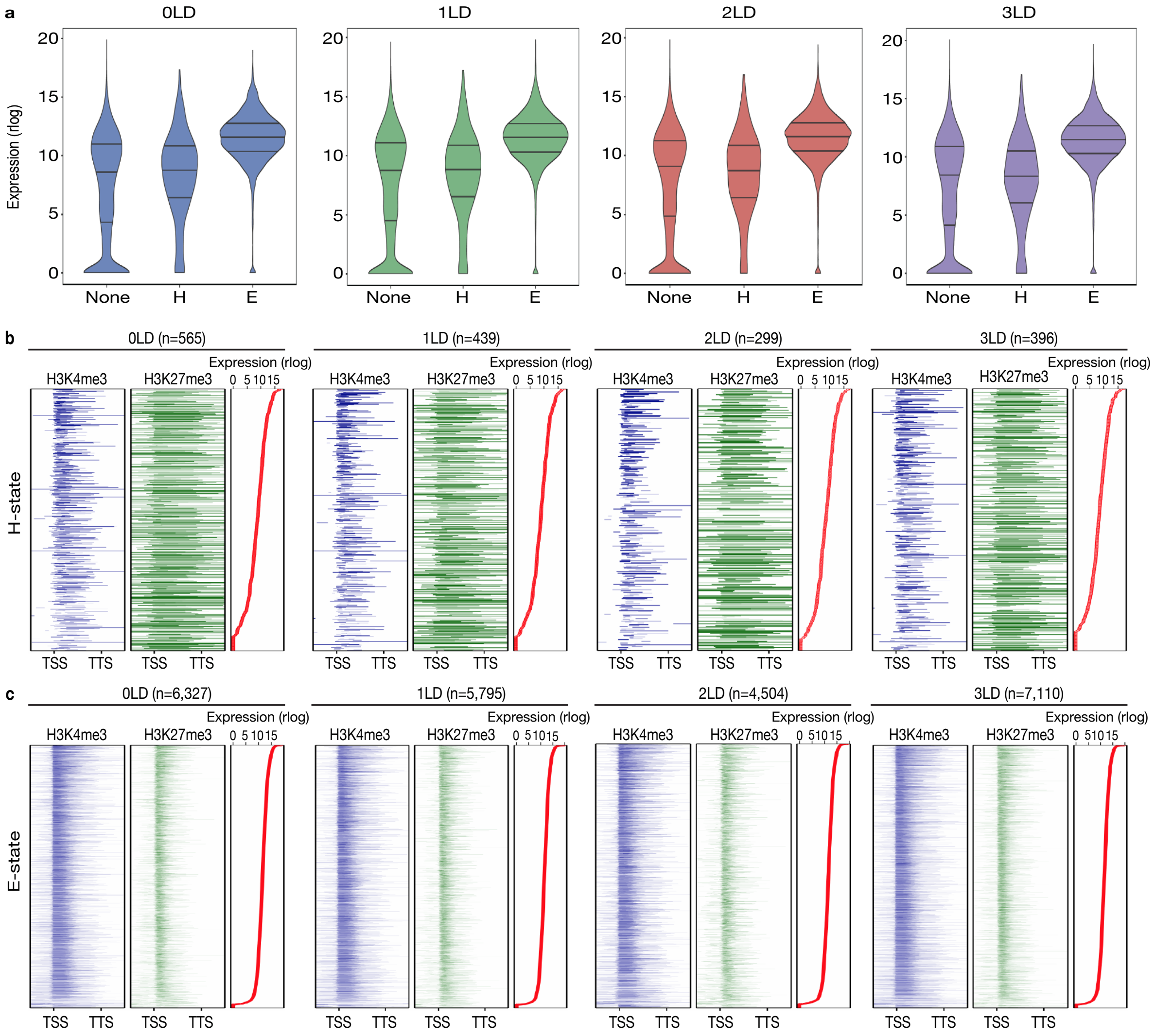

Supplementary Figure 10. Profiles of genes in H- and E-state during floral transition a. Distribution of expression of genes in H-state, E-state, or neither state (None) at different time points. **b**, Distribution of H3K4me3 and H3K27me3 marks across the gene body (±1 kb) of H-state genes in meristems at different time points. Genes of each state were ordered by expression level in the meristem represented by red lines. **c**, Distribution of H3K4me3 and H3K27me3 marks across the gene body (±1 kb) of E-state genes in meristems at different time points. Genes of each state were ordered by expression level [regularized logarithm (rlog) of counts] in the meristem represented by red lines.

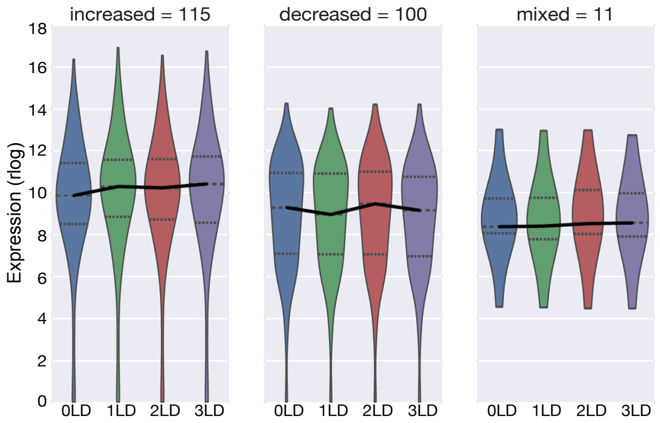

Supplementary Figure 11. Distribution of expression [regularized logarithm (rlog) of counts] of transcription factor genes that are differentially H3K4me3-modified. Panels display the distribution of expression of genes with increased (n=115), decreased (n=100), and mixed (n=11) changes in H3K4me3 at the four time points analyzed [blue: 0LD (=SD); green: 1LD; red: 2LD; purple: 3LD]. The median of expression is shown by a bold line.

Supplementary Table 1. Sequences of oligonucleotide primers used in this work

Gene	Oligo	Sequence (5' -> 3')	Comment
		**I. Oligonucleotides used for cloning**	
BirA	G-30593	ATGAAGGATAACACCGTG	966 bp (*E. coli* biotin ligase)
	G-30594	TTATTTTTCTGCACTACGCA	
redNTF	G-28797	ATGAATCATTCAGCGAAAACCA	333 bp (WPP domain)
	G-28798	CTCACAGCTGCTGCCTCAACCTC	
	G-28799	TGAGGCAGCAGCTGTGAGCAAGGGCGAGGAG	705 bp (mCherry ORF)
	G-28800	AGCAGCAGCAGCAGCCTTGTACAGCTCGTCCATGC	
	G-28801	AAGGCTGCTGCTGCTGCTATGGCTGGTGGAC	69 bp (BLRP domain)
	G-33192	ACTGGATCCTCAAGATCCACCAGTATC	
	G-33191	AGTTCATCTACAAGGTGAAGCTGC	428 bp (NTF *in situ* probe)
	G-33192	ACTGGATCCTCAAGATCCACCAGTATC	
pAt3g59270	G-26962	CGGTCGACGACCTTACTCTTTTTGTTTTC	1393 bp
	G-26963	GGAACATGCTCCCGGGCTTTTTCAAATGCAAATCAC	
tAt3g59270	G-26964	CATTTGAAAAAGCCCGGGAGCATGTTCCCTTTTCATCGGC	1404 bp
	G-27201	CGCACTAGTAGAGTTTGAAGCAATAACCAG	
		**II. Oligonucleotides used for RT-PCR**	
STM	G-26769	GGATAATAGTGATGGTCCGA	176 bp
	G-26770	GGGGAGGAGCTAGTGATTGA	
TUB2	N-0078	GAGCCTTACAACGCTACTCTGTCTGTC	167 bp
	N-0079	ACACCAGACATAGTAGCAGAAATCAAG	
SOC1	G-30998	AAACGAGAAGCTCTCTGAAAAG	142 bp
	G-30999	AAGAACAAGGTAACCCAATGAAC	
CLV3	G-11464	GATGAAAATGGAAAGTGAATGG	165 bp
	G-11465	GGGAGCTGAAAGTTGTTTCTTG	
FD	G-30964	GCAAGACTCAAGAGACAACAAG	98 bp
	G-30965	CAAAATGGAGCTGTGGAAGAC	
AGL24	G-30958	GTCAAGCTACGACAGTGGAAC	119 bp
	G-30959	CATCGAAGTCAACTCTGCTG	

Supplementary Table 2. RNA-seq and ChIP-seq summary statistics

Tissue	Time	Rep.	Antibody	Reads	AR [%]	UAR [%]	Comment
**I RNA-seq**							
Meristem	0 LD	1	n/a	6.244.794			a
		2	n/a	7.383.714			a
		3	n/a	8.634.136			b
		4	n/a	6.429.387			b
	1 LD	1	n/a	7.388.182			a
		2	n/a	6.676.964			a
		3	n/a	8.411.545			b
		4	n/a	8.247.744			b
	2 LD	1	n/a	7.590.875			a
		2	n/a	8.482.348			a
		3	n/a	6.568.625			b
		4	n/a	7.687.822			b
	3 LD	1	n/a	7.551.584			a
		2	n/a	6.559.382			a
		3	n/a	10.778.410			b
		4	n/a	11.277.106			b
**II. ChIP-seq**							
Meristem	0 LD	1	H3	22.986.538	86,19	61,17	c
			H3K4me3	20.027.626	72,78	66,86	c
			H3K27me3	50.101.780	57.04	51.54	c
		2	H3	26.851.335	87,80	62,53	c
			H3K4me3	24.489.295	72,32	67,78	c
			H3K27me3	22.606.306	62,43	55,72	c
	1 LD	1	H3	26.358.528	76,89	54,55	c
			H3K4me3	28.859.434	39,83	36,85	c
			H3K27me3	94.014.073	24.76	21.31	c
		2	H3	21.618.155	84,19	59,42	c
			H3K4me3	19.321.126	60,87	57,50	c
			H3K27me3	25.555.702	49,89	44,69	c
	2 LD	1	H3	29.937.923	81.88	57.36	c
			H3K4me3	19.281.779	42,46	39,37	c
			H3K27me3	58.644.112	36.63	30.93	c
		2	H3	26.743.633	73,00	51,09	c
			H3K4me3	25.053.466	52,26	48,66	c
			H3K27me3	39.104.365	36.75	32.67	c
	3LD	1	H3	24.328.989	87,68	59,75	c
			H3K4me3	24.302.843	62,11	58,71	c
			H3K27me3	23.197.303	67,96	57,31	c
		2	H3	26.667.467	77,67	54,53	c
			H3K4me3	27.213.213	60,89	56,49	c
			H3K27me3	28.151.158	62,23	54,36	c
Seedling	21 LD	1	H3	87.247.688	89,47	74,65	d
			H3K4me3	35.893.834	96,86	94,49	d
			H3K27me3	36.177.266	96,27	91,54	d
		2	H3	87.247.688	89,88	74,93	d
			H3K4me3	35.893.834	96,86	94,54	d
			H3K27me3	36.177.266	96,18	92,35	d

AR [%] = aligment rate [%]; UAR [%] = unique aligment rate [%]; Comments: a) Hiseq 2000 system, 2×101 base pair paired-end; b) Hiseq 3000 system, 2×150 base pair paired-end (trimmed to 2 x 101 bp); c) Hiseq 2000 and Miseq, 50 base pair single-end; d) Hiseq 3000, 150 base pair paired-end (only the first 50bp of the first read were used).
